# Facile preparation and conversion of 4,4,4-trifluorobut-2-yn-1-ones to aromatic and heteroaromatic compounds

**DOI:** 10.3762/bjoc.17.14

**Published:** 2021-01-15

**Authors:** Takashi Yamazaki, Yoh Nakajima, Minato Iida, Tomoko Kawasaki-Takasuka

**Affiliations:** 1 Division of Applied Chemistry, Institute of Engineering, Tokyo University of Agriculture and Technology, 2-24-16 Nakamachi, Koganei 184-8588, Japan

**Keywords:** CF_3_-containing propargylic alcohols, CF_3_-containing ynones, oxidation, pyrimidines, salicylate derivatives

## Abstract

The concise preparation of 4,4,4-trifluorobut-2-yn-1-ones by the oxidation of the readily accessible corresponding propargylic alcohols as well as their utilization as Michael acceptors for the construction of aromatic and heteroaromatic compounds are reported.

## Introduction

It is well known that the incorporation of a fluorine atom or a fluorine-containing group into organic compounds sometimes affects their original biological activities [1–3], and thus the development of methods for the construction of such molecules with this atom or group is an important task. During our research in the field of fluorine chemistry, we have previously developed methods to get convenient access to CF_3_-containing propargylic alcohols **1** using 2-bromo-3,3,3-trifluoropropene [4–5] as well as 1-chloro-3,3,3-trifluoropropene [6] as a substrate and to utilize **1** by way of a variety of routes [7–14]. Recently, we turned our attention to oxidized propargyl alcohols, namely ynones **2**, because of the interesting structure with two strongly electron-withdrawing moieties, resulting in a high electrophilicity. However, only a few methods have been reported thus far for their preparation [15–20], including one that appeared in 2020 [21]. However, they suffer from problems, such as the formation of the desired product **2** only as regioisomeric mixtures [15,17] or the applicability to only one substrate without any extension to other related compounds [16,18–20]

Aromatic and heteroaromatic compounds are recognized as useful intermediates, and as pointed out above, the expected high electrophilicity of **2** was considered to allow the utilization as efficient Michael acceptors. Actually, this was the case, and the Sandford group clarified the high potency of these compounds for the conjugate addition of N- as well as O-nucleophiles [21]. On the basis of such an idea, we tried two routes to gain access to 1) 4-substituted 6-(trifluoromethyl)salicylate derivatives (C-nucleophiles) and 2) 6-substituted 4-(trifluoromethyl)pyrimidines (N-nucleophiles), both in a concise fashion, with the full details, including the preparation of **2**, being reported herein.

## Results and Discussion

Investigations on the reaction conditions were carried out for the oxidation of propargylic alcohols **1**, which were readily accessible by our already reported procedure [4,19] as well as by the ones developed by other groups [22–23]. In spite of the previous unfavorable results for the oxidative transformation of **1a** by MnO_2_ as well as other oxidants [18,23], MnO_2_ was selected due to the low cost and convenient handling. After a number of trials aimed at optimizing the reaction conditions, the unexpectedly smooth conversion of **1a** to **2a** was realized. Although it was reported [18,23] that only a small amount of **2a** was confirmed after the reaction of **1a** with 10 equiv of MnO_2_ at ambient temperature after 1 day, the same amount of oxidant actually led to the complete conversion after only 4 h at room temperature. In this instance, for the clean preparation of the desired compound **2a**, the important point was to initiate this oxidation process at 0 °C, followed by warming to ambient temperature. With this intriguing result in hand, the oxidation of the other substrates **1** was performed under similar reaction condition, the results of which are summarized in Table 1.

**Table 1:** Oxidation of propargylic alcohols **1**.^a^

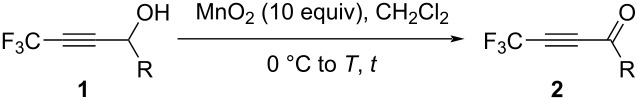					

entry	R	*T* (°C)	*t* (h)	product	^19^F NMR yield^b^ (%)

1	Ph	rt	4	**2a**	95 (100)
2	4-(MeO)C_6_H_4_	rt	2.5	**2b**	93 (100)
3	4-MeC_6_H_4_	rt	3	**2c**	90 (100)
4	4-BrC_6_H_4_	rt	2	**2d**	58 (93)
5	PhCH_2_CH_2_	reflux	20	**2e**	35 (48)
6^c^	PhCH_2_CH_2_	reflux	20	**2e**	25 (80)

^a^**1** was added to a CH_2_Cl_2_ solution of MnO_2_ at 0 °C, and the mixture was stirred with gradually warming to a defined temperature for a defined reaction time, as indicated. ^b^The conversion of **1** as determined by ^19^F NMR spectroscopy is shown in parentheses. ^c^CHCl_3_ was used instead of CH_2_Cl_2_.

We reported the yield as determined by ^19^F NMR spectroscopy rather than after purification because of the inherent instability of **2** on silica gel, causing partial decomposition. The crude mixture proved to be substantially pure, and thus **2c** and **2d** afforded appropriate analytical data, including NMR (^1^H, ^13^C, and ^19^F), IR, as well as high-resolution MS data. This process allowed us to obtain the required ynones **2** as long as the residue R was aromatic, while with an aliphatic substituent in this position, a full conversion was not observed even after a prolonged reaction period and at a higher temperature, and such conditions seemed to evoke the degradation of the products **2** to some extent. This was not the case for the Dess–Martin periodinane oxidation and the propargylic alcohols with C_7_H_15_ as the residue R^1^ by Hoye et al. [15] and C_9_H_19_ as the residue R^1^ by the Sandford group [21], who recorded 80% and 76% isolated yield, respectively, of the corresponding ketones. Because these compounds were previously isolated inconveniently by preparative GLC [15] or distillation [17,19], we decided to employ them without further purification.

The Michael addition reactions were then carried out under basic conditions for the ynone **2a** thus obtained, which, to our astonishment, was found to be relatively rare even for the nonfluorinated counterparts (Table 2) [24–28].

**Table 2:** Michael addition of acetylacetone to **2a**.

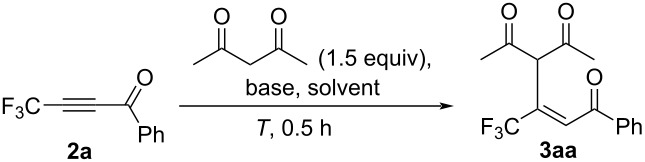				

entry	base (equiv)	solvent	*T* (°C)	^19^F NMR yield^a^ (%)

1	NaH (1.5)	THF	0	53
2	NaH (1.5)	DMF	0	34
3	NaH (1.5)	Et_2_O	0	76 (69)
4	NaH (1.5)	Et_2_O	−40	72
5	NaH (1.5)	Et_2_O	rt	61
6	*t*-BuOK (1.5)	Et_2_O	0	68 (67)
7	*t*-BuOK (0.1)	Et_2_O	0	77
8	*t*-BuOK (0.02)	Et_2_O	0	94 (84)
9	*t*-BuOK (0.02)	*t*-BuOH	30	78
10	DBU (0.1)	Et_2_O	0	33
11	TMG^b^ (0.1)	Et_2_O	0	14

^a^The isolated yield is shown in parentheses. ^b^1,1,3,3-Tetramethylguanidine.

As expected for this model system, a very efficient addition was observed within a period of 0.5 h at 0 °C, leading to an isolated yield of 69% for the adduct **3aa** when 1.5 equiv of acetylacetone and NaH in Et_2_O were used (Table 2, entries 1−3), which unambiguously demonstrated the high potency of **2a** as a Michael acceptor. Stronger bases, such as NaH and *t*-BuOK seemed to be preferable (Table 2, entries 3 and 6), and amines such as DBU and 1,1,3,3-tetramethylguanidine (TMG) were found to be insufficient (Table 2, entries 10 and 11). Judging from the possible mechanism, this process was considered to proceed under the action of a catalytic amount of a base, and only 2 mol % of *t*-BuOK led to the formation of the desired adduct **3aa** as a sole stereoisomer in 84% isolated yield under the same reaction conditions (Table 2, entries 6−8). The *E*-stereochemistry of **3aa** was deduced from the NMR data of structurally similar compounds [29] on the basis of 1) comparison of the chemical shift of the CF_3_ group and 2) the absence of a coupling between the vinylic and allylic protons. In order to explore other nucleophiles, we employed 1,3-diketones, such as 5-methylhexane-1,3-dione and 5,5-dimethylhexane-1,3-dione (that is, one of the two methyl groups in acetylacetone was formally substituted for an isopropyl or a *t*-Bu moiety, respectively), but no reaction was observed at all. The conjugate addition of amines to this type of ynone has already been reported [21], where the authors pointed out the significant slowdown of the reaction rate when *tert*-butylamine was employed, which was considered to be a reflection of the sensitivity of ynones **2** towards steric hindrance.

Because appropriate conditions for the initial Michael addition reactions were thus determined, further conversion to the benzene derivative **4aa** was also performed (Table 3).

**Table 3:** Investigation of the cyclization conditions for **3aa**.

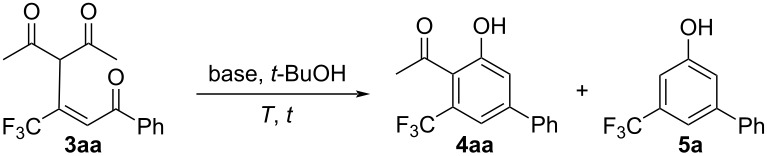				

entry	base (equiv)	*T* (°C)	*t* (h)	^19^F NMR yield of **4aa**^a^ (%)

1	*t*-BuOK (0.5)	60	4	7 (49)
2	*t*-BuOK (1.0)	60	4	13 (81)^b^ {8}
3	*t*-BuOK (2.0)	60	4	85 (<1)
4	*t*-BuOK (3.0)	60	4	>99 (0)
5	*t*-BuOK (3.0)	60	2	>99 (0)
6	*t*-BuOK (3.0)	30	2	>99 (0) {84}
7^c^	*t*-BuOK (3.0)	30	2	>99 (0)
8	*t*-BuONa (3.0)	30	2	58 (0)
9	*t*-BuOLi (3.0)	30	3	66 (3)
10^d^	EtONa (3.0)	30	2	0 (0)
11^d^	EtONa (3.0)	60	6	18 (15)

^a^The ^19^F NMR yield of **5a** and the isolated yield of **4aa** are shown in parentheses and braces, respectively. ^b^Isolated yield of **5a**. ^c^Et_2_O was used instead of *t*-BuOH. ^d^EtOH was used instead of *t*-BuOH.

First of all, **3aa** was introduced to a *t*-BuOH solution of 2 equiv of *t*-BuOK to afford the desired product **4aa** in a high yield, and an increase in the amount of this base to 3 equiv led to the complete conversion of the substrate **3aa** (Table 3, entries 3 and 4). This trend was followed even when the temperature was decreased to 30 °C and the reaction time was 2 h, furnished **4aa** in a 84% yield (Table 3, entries 6 and 7). The use of the corresponding lithium and sodium *tert*-butoxides was found to afford a lower yield, which clarified the importance of the ionic character of the base (Table 3, entries 8 and 9).

**Scheme 1:**
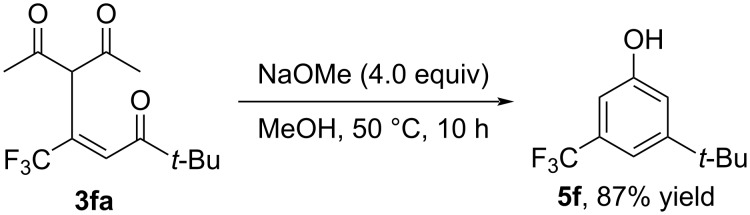
Deacetylative cyclization of **3fa**.

For the present transformation, Hu and Guan already reported a similar type of ring closure of the substrate **3fa** [30], which allowed the conversion to the phenolic product **5f** as a result of the cyclization and was accompanied by deacetylation under the conditions depicted in Scheme 1 [31–33].

In our case, it was quite interesting to note that the deacylated product **5a** was also obtained as the major product when an equimolar or lesser amount of *t*-BuOK was employed (Table 3, entries 1 and 2), along with formation of a minor amount of **4aa**. Moreover, when the isolated product **4aa** was subjected to the conditions shown in Table 3, entry 4, no reaction was observed at all, with recovery of **4aa**, which clearly demonstrated that the deacetylation step had occurred before the conversion to **4aa**. In the present cyclization, the ionic character of the alkoxide seemed to be an important factor, and the corresponding lithium and sodium salts did not aid the reaction satisfactory. Moreover, EtONa was found to be inappropriate for this protocol (Table 3, entries 10 and 11).

Determination of the appropriate conditions both for Michael addition as well as cyclization/aromatization reactions allowed us to explore a brief scope and limitation of the present reaction by using four representative types of crude ynones **2** starting from the conjugate addition step either with acetylacetone or ethyl acetoacetate (Table 4).

**Table 4:** Preparation of the trifluoromethylated salicylate derivatives **4**.

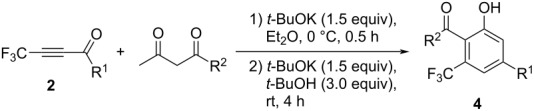				

entry	R^1^	R^2^	product	isolated yield (%)

1	Ph	Me	**4aa**	74
2	Ph	EtO	**4ab**	80
3	4-(MeO)C_6_H_4_	Me	**4ba**	67
4	4-(MeO)C_6_H_4_	EtO	**4bb**	73
5	4-Me-C_6_H_4_	Me	**4ca**	68
6	4-Me-C_6_H_4_	EtO	**4cb**	75
7	4-Br-C_6_H_4_	Me	**4da**	70
8	4-Br-C_6_H_4_	EtO	**4db**	54

In all instances, the desired products **4** were obtained in good to excellent yield without contamination by the deacetylated products **5**.

With the success of the regiospecific formation of 4-substituted 6-(trifluoromethyl)salicylate derivatives **4**, as shown in Table 4, the application of ynones **2** was demonstrated at the next stage for the construction of a variety of pyrimidine derivatives, **6**, using amidines including guanidine [34]. First of all, various bases were employed for the reaction of the model substrate **2a**. During initial testing, guanidine hydrochloride at 25 °C for 4 h in acetonitrile (Table 5, entries 1–7) as well as sodium carbonate were found to be adequate (Table 5, entry 5).

**Table 5:** Preparation of trifluoromethylated pyrimidine derivatives **6aa**.

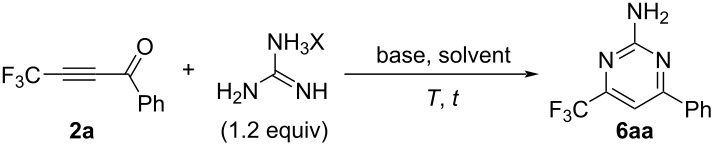						

entry	base	solvent	*T* (°C)	*t* (h)	^19^F NMR yield^a^ (%)	
					**6aa**	byproducts

1	KO*t*-Bu	CH_3_CN	25	4	8	54
2	NaOEt	CH_3_CN	25	4	15	9
3	DBU	CH_3_CN	25	4	3	17
4	K_2_CO_3_	CH_3_CN	25	4	28	4
5	Na_2_CO_3_	CH_3_CN	25	4	33	17
6	CaCO_3_	CH_3_CN	25	4	25	39
7	Li_2_CO_3_	CH_3_CN	25	4	27	29
8	Na_2_CO_3_	DME	25	4	19	24
9	Na_2_CO_3_	DMF	25	4	31	14
10	Na_2_CO_3_	DMSO	25	4	24	9
11	Na_2_CO_3_	THF	25	4	31	20
12	Na_2_CO_3_	CH_3_CN	50	4	66	5
13	Na_2_CO_3_	CH_3_CN	80	4	81 (57)	2
14	Na_2_CO_3_	CH_3_CN	80	8	83 (60)	0
15	Na_2_CO_3_	CH_3_CN	80	16	70 (56)	<1

^a^The isolated yield is shown in parentheses.

A comparison of the results in Table 5, entries 8–11 vs entry 5 led to the determination that the adequate solvent was acetonitrile. After screening the reaction time and temperature (Table 5, entries 12–15 vs entry 5), we eventually selected the conditions as shown in Table 5, entry 14, which included heating the reaction mixture at 80 °C for 8 h in MeCN, furnishing the desired pyrimidine **6aa** in 60% isolated yield.

Then, four types of ynones, **2a**–**d**, were reacted with formamidine and acetamidine as well as guanidine under the optimized conditions outlined above, the results of which are summarized in Table 6. When the reactions were performed with acetamidine and guanidine, a good to high yield was recorded, while only a lower yield was obtained by the application of formamidine acetate, for which the reaction was not further elucidated at this point.

**Table 6:** Preparation of trifluoromethylated pyrimidine derivatives **6**.

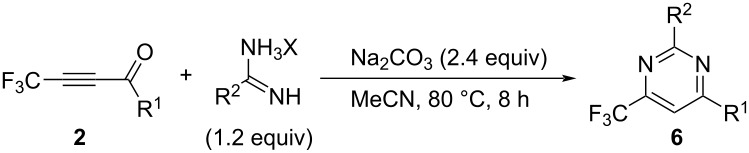					

entry	R^1^	R^2^	X	product	isolated yield (%)

1	Ph (**2a**)	NH_2_	Cl	**6aa**	60
2	4-(MeO)C_6_H_4_ (**2b**)	NH_2_	Cl	**6ab**	55
3	4-MeC_6_H_4_ (**2c**)	NH_2_	Cl	**6ac**	52
4	4-BrC_6_H_4_ (**2d**)	NH_2_	Cl	**6ad**	67
5	Ph (**2a**)	Me	Cl	**6ba**	67
6	4-(MeO)C_6_H_4_ (**2b**)	Me	Cl	**6bb**	75
7	4-MeC_6_H_4_ (**2c**)	Me	Cl	**6bc**	53
8	4-BrC_6_H_4_ (**2d**)	Me	Cl	**6bd**	57
9	Ph (**2a**)	H	AcO	**6ca**	21
10	4-(MeO)C_6_H_4_ (**2b**)	H	AcO	**6cb**	28
11	4-MeC_6_H_4_ (**2c**)	H	AcO	**6cc**	22
12	4-BrC_6_H_4_ (**2d**)	H	AcO	**6cd**	26

The unfavorable yield may be reasoned by the fact that there have been very limited reports for the syntheses of, for example, pyrimidine **6ca** [35–38], with such drawbacks as the formation of a regioisomeric mixture [35] and the requirement of harsh conditions [38]. At the same time, for the use of a substrate similar to **2a** where the CF_3_ and Ph groups were formally exchanged, an isolated yield of 84% has been recorded for **6ca** [37]. Moreover, it is noteworthy that only one report dealt with the preparation of **6cb** [37], and **6cc** and **6cd** have not been previously reported. Thus, including **6aa** [37–40] and **6ba** [37–38,41–42], it is noteworthy to realize the construction of relatively rare pyrimidines in a concise fashion via the ynones **2** with a CF_3_ group.

## Conclusion

As described above, we succeeded in the construction of 4,4,4-trifluorobut-2-yn-1-ones **2** by way of a convenient MnO_2_ oxidation of readily accessible propargylic alcohols **1**. In spite of the application of this method only to **1**, with an aromatic substituent at the propargylic position and the requirement of an excess amount of this oxidant, the practical convenience, low cost, as well as the clean conversion are notable features of this method, which is worthy of attention. Furthermore, the high electrophilicity of ynones **2** obtained was nicely demonstrated through two representative reactions, namely 1) Michael additions of β-keto carbonyl compounds and 2) amidines and guanidine for the construction of aromatic and heteroaromatic products, which successfully expanded this unique synthetic approach to compounds with a limited precedent. Further work is ongoing for the application of ynones **2**.

## Supporting Information

The Supporting Information features the experimental part of this article.

File 1 General information, synthetic procedures, and spectral data.

## References

[1] Inoue M, Sumii Y, Shibata N (2020). ACS Omega.

[2] Mei H, Han J, Fustero S, Medio‐Simon M, Sedgwick D M, Santi C, Ruzziconi R, Soloshonok V A (2019). Chem – Eur J.

[3] Wang J, Sánchez-Roselló M, Aceña J L, del Pozo C, Sorochinsky A E, Fustero S, Soloshonok V A, Liu H (2014). Chem Rev.

[4] Mizutani K, Yamazaki T, Kitazume T (1995). J Chem Soc, Chem Commun.

[5] Yamazaki T, Mizutani K, Kitazume T (1995). J Org Chem.

[6] Miyagawa A, Naka M, Yamazaki T, Kawasaki-Takasuka T (2009). Eur J Org Chem.

[7] Yamazaki T, Ichige T, Kitazume T (2004). Org Lett.

[8] Yamazaki T, Yamamoto T, Ichihara R (2006). J Org Chem.

[9] Yamazaki T, Kawasaki-Takasuka T, Furuta A, Sakamoto S (2009). Tetrahedron.

[10] Watanabe Y, Yamazaki T (2009). Synlett.

[11] Watanabe Y, Yamazaki T (2010). J Fluorine Chem.

[12] Watanabe Y, Yamazaki T (2011). J Org Chem.

[13] Yamazaki T, Watanabe Y, Yoshida N, Kawasaki-Takasuka T (2012). Tetrahedron.

[14] Ichikawa T, Kawasaki-Takasuka T, Yamada S, Yamazaki T, Kubota T (2013). J Fluorine Chem.

[15] Shen Y, Xin Y, Cen W, Huang Y (1984). Synthesis.

[16] Bumgardner C L, Bunch J E, Whangbo M H (1986). J Org Chem.

[17] Chechulin P I, Filyakova V I, Pashkevich K I (1989). Bull Acad Sci USSR, Div Chem Sci (Engl Transl).

[18] Tajammal S, Tipping A E (1990). J Fluorine Chem.

[19] Brisdon A K, Crossley I R (2002). Chem Commun.

[20] Wang T, Niu D, Hoye T R (2016). J Am Chem Soc.

[21] Murray B J, Marsh T G F, Yufit D S, Fox M A, Harsanyi A, Boulton L T, Sandford G (2020). Eur J Org Chem.

[22] Ishihara T, Yamasaki Y, Ando T (1985). Tetrahedron Lett.

[23] Sibous L, Tipping A E (1993). J Fluorine Chem.

[24] Fouli F A, Basyouni M N (1981). Acta Chim Acad Sci Hung.

[25] Shankar R, Jha A K, Singh U S, Hajela K (2006). Tetrahedron Lett.

[26] Ramachary D B, Venkaiah C, Murali Krishna P (2012). Chem Commun.

[27] Tong W, Li Q-Y, Xu Y-L, Wang H-S, Chen Y-Y, Pan Y-M (2017). Adv Synth Catal.

[28] Yuan Y, Guo Z, Mu Y, Wang Y, Xu M, Li Y (2020). Adv Synth Catal.

[29] Ramasamy M, Lin H-C, Kuo S-C, Hsieh M-T (2019). Synlett.

[30] Guan H-P, Hu C-M (1996). J Fluorine Chem.

[31] Furuta T, Nakayama M, Suzuki H, Tajimi H, Inai M, Nukaya H, Wakimoto T, Kan T (2009). Org Lett.

[32] Wang X-j, Zhang L, Byrne D, Nummy L, Weber D, Krishnamurthy D, Yee N, Senanayake C H (2014). Org Lett.

[33] Matsuda T, Matsumoto T (2016). Org Biomol Chem.

[34] Nenajdenko V G (2014). Fluorine in Heterocyclic Chemistry.

[35] Gupta S, Melanson J A, Vaillancourt L, Nugent W A, Tanoury G J, Schatte G, Snieckus V (2018). Org Lett.

[36] Geri J B, Wade Wolfe M M, Szymczak N K (2018). Angew Chem, Int Ed.

[37] Romanov A R, Rulev A Y, Ushakov I A, Muzalevskiy V M, Nenajdenko V G (2017). Eur J Org Chem.

[38] Saijo R, Watanabe G, Kurihara K-i, Kawase M (2014). Heterocycles.

[39] Liu C, Cui Z, Yan X, Qi Z, Ji M, Li X (2016). Molecules.

[40] Rawal R K, Tripathi R, Katti S B, Pannecouque C, De Clercq E (2007). Bioorg Med Chem.

[41] Funabiki K, Nakamura H, Matsui M, Shibata K (1999). Synlett.

[42] Jeong I H, Jeon S L, Kim M S, Kim B T (2004). J Fluorine Chem.

