# Supplementary material for: Facile preparation and conversion of 4,4,4-trifluorobut-2-yn-1-ones to aromatic and heteroaromatic compounds
Source: Beilstein J Org Chem. 2021 Jan 15;17:132–8. doi: 10.3762/bjoc.17.14 (PMC7814177; doi:10.3762/bjoc.17.14)
Supplement: File 1 — General information, synthetic procedures, and spectral data. [file Beilstein_J_Org_Chem-17-132-s001.pdf]

BEILSTEIN JOURNAL OF ORGANIC CHEMISTRY

Supporting Information

for

Facile preparation and conversion of 4,4,4-trifluorobut-2-yn-1-ones to aromatic and heteroaromatic compounds

Takashi Yamazaki, Yoh Nakajima, Minato Iida and Tomoko Kawasaki-Takasuka

Beilstein J. Org. Chem. **2021,** *17,* 132–138. doi:10.3762/bjoc.17.14

General information, synthetic procedures, and spectral data

License and Terms: This is a supporting information file under the terms of the Creative Commons Attribution License (https://creativecommons.org/licenses/by/4.0). Please note that the reuse, redistribution and reproduction in particular requires that the author(s) and source are credited and that individual graphics may be subject to special legal provisions.
The license is subject to the *Beilstein Journal of Organic Chemistry* terms and conditions: (https://www.beilstein-journals.org/bjoc/terms)

Table of contents

1. General information S2

2. Synthetic procedure and characterization of new compounds S2

  2.1. General procedure for the oxidation of the propargylic alcohols S2

  2.2. General procedure for the Michael addition reactions S3

  2.3. General procedure for the cyclization to phenol compounds S4

  2.4. 3-Phenyl-5-(trifluoromethyl)phenol S6

  2.5. General procedure for the preparation of the trifluoromethylated pyrimidines S6

3. Spectral data S10

4. References S36

1. General information

Most of the reactions where an organic solvent was employed were performed under argon, with magnetic stirring using flame-dried glassware. Unless otherwise noted, the materials were obtained from commercial suppliers, including anhydrous THF, Et_2O, and CH_2Cl_2 and were used without further purification. DMSO was freshly dried prior to the reaction over 4 Å molecular sieves, which were activated by irradiating with a microwave for 1 min and heating under vacuum for 1 h. Analytical thin-layer chromatography (TLC) was routinely used for monitoring reactions by generally using a mixture of hexane and ethyl acetate. Spherical neutral silica gel (63–210 μm) was employed for usual column chromatography.

^{1}H (300.40 MHz), ^{13}C (75.45 Hz), and ^{19}F NMR(282.65 Hz) spectra were recorded in $CDCl_3$ unless otherwise noted, and chemical shifts were reported in parts per million (ppm), downfield from internal tetramethylsilane (Me_4Si: δ 0.00, for ^{1}H and ^{13}C) or hexafluorobenzene (C_6F_6: δ −163.00 for ^{19}F). Data are tabulated in the following order: number of protons or fluorine atoms, multiplicity (s, singlet; d, doublet; t, triplet; q, quartet; quint, quintet; sex, sextet; sept, septet; m, multiplet; b, broad peak), coupling constants in Hertz. For ^{13}C NMR, because it is difficult to observe perfluoroalkyl carbon atoms even after long-time data acquisition due to multiple coupling, these data are not shown. Infrared (IR) spectra are reported in wave numbers (cm^{-1}). High-resolution mass spectrometry was performed in the positive ionization mode. Melting points were measured by differential scanning calorimetry (DSC; using a Shimadzu DSC-60 device).

2. Synthetic procedure and characterization of new compounds

2.1. General procedure for the oxidation of the propargylic alcohols

2.1.1. 4,4,4-Trifluoro-1-phenylbut-2-yn-1-one (2a) [1]

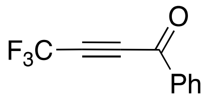

To a 100 mL two-necked round-bottomed flask containing 9.493 g of MnO_2 (10.92 mmol) and CH_2Cl_2 (50 mL) at 0 °C under an argon atmosphere was added 1.31 g of 4,4,4-trifluoro-1-phenylbut-2-yn-1-ol (**1a**, 6.54 mmol) [2], and stirring was continued for 4 h with removing the ice bath. Filtration with Celite®, followed by evaporation of the volatiles afforded a crude mixture in 95% yield determined by ^{19}F NMR spectroscopy, which was found to be unstable towards silica gel and used afterward without further purification. Rf 0.65 (Hexane:AcOEt=4:1). ^{1}H NMR δ 7.56 (t, *J*=8.0 Hz, 2H), 7.72 (tt, *J*=7.5, 1.5 Hz, 1H), 8.11 (td, *J*=7.2, 1.9 Hz, 2H). ^{13}C NMR δ 75.0 (q, *J*= 54.2 Hz), 79.9 (q, *J*=6.4 Hz), 113.8 (q, *J*=259.6 Hz), 129.0, 129.7, 135.0, 135.5, 174.8. ^{19}F NMR δ –52.84 (s).

2.1.2. 4,4,4-Trifluoro-1-(4-methoxyphenyl)but-2-yn-1-one (2b) [1]

The reaction of 4,4,4-trifluoro-1-(4-methoxyphenyl)but-2-yn-1-ol (**1b**) [3]and MnO_2 was

continued for 2.5 h to afford the crude product in 93% ^{19}F NMR yield as a red solid. mp 40–41 °C. Rf 0.50 (Hexane: AcOEt=4:1). ^{1}H NMR δ 3.93 (s, 3H), 7.01 (d, *J*=9.0 Hz, 2H), 8.08 (d, *J*=9.0 Hz, 2H). ^{13}C NMR δ 55.7, 74.7 (q, *J*=54.2 Hz), 80.3 (q, *J*=6.4 Hz), 113.9 (q, *J*=259.5 Hz), 114.4, 128.5, 132.3, 165.6, 173.2. ^{19}F NMR δ –52.71 (s).

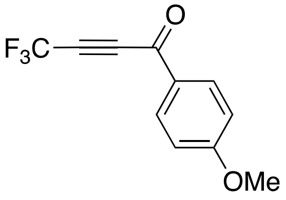

2.1.3. 4,4,4-Trifluoro-1-(4-methylphenyl)but-2-yn-1-one (2c)

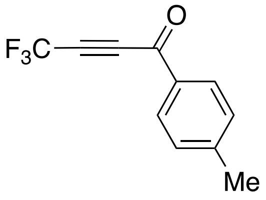

The reaction of 4,4,4-trifluoro-1-(4-methylphenyl)but-2-yn-1-ol (**1c**) [4] and MnO_2 was continued for 3 h to afford the crude product in 90% ^{19}F NMR yield as a white oil. Rf 0.57 (Hexane:AcOEt=4:1). ^{1}H NMR δ 2.47 (s, 3H), 7.34 (d, *J*=8.7 Hz, 2H), 7.99 (d, *J*=8.4 Hz, 2H). ^{13}C NMR δ 21.6, 74.6 (q, *J*=53.9 Hz), 80.1 (q, *J*=6.2 Hz), 113.8 (q, *J*=259.2 Hz), 129.7, 129.8, 132.8, 147.1, 174.3. ^{19}F NMR δ –53.78 (s). IR (neat) ν 3301, 3037, 2927, 1661, 1605, 1573, 1450, 1412, 1269, 1153 cm^{-1}. HRMS-FAB (*m*/*z*): $[M+H]^+$ calcd. for $C_{11}H_8F_3O$, 213.0527; found, 213.0520.

2.1.4. 1-(4-Bromophenyl)-4,4,4-trifluorobut-2-yn-1-one (2d)

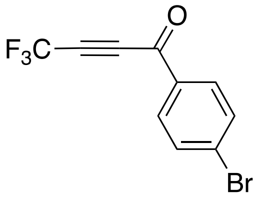

The reaction 1-(4-bromophenyl)-4,4,4-trifluorobut-2-yn-1-ol (**1d**) [5] and MnO_2 was continued for 2 h to afford the crude product in 58% ^{19}F NMR yield as a yellow solid. mp 60–61 °C. Rf 0.69 (Hexane: AcOEt=10:1). ^{1}H NMR δ 7.71 (d, *J*=8.7 Hz, 2H), 7.96 (d, *J*=8.7 Hz, 2H). ^{13}C NMR δ 75.7 (q, *J*=55.2 Hz), 79.5 (q, *J*=6.2 Hz), 113.7 (q, *J*=259.8 Hz), 131.0, 131.4, 132.5, 133.9, 174.0. ^{19}F NMR δ –52.86 (s). IR (KBr) ν 3291, 3090, 1662, 1587, 1486, 1401, 1155, 1073, 1011, 838 cm^{-1}. HRMS-FAB (m/z): $[M+H]^+$ calcd. for $C_{10}H_5{}^{79}BrF_3O$, 276.9476; found, 276.9478. $[M+H]^+$ calcd. for $C_{10}H_5{}^{81}BrF_3O$, 278.9455; found, 278.9465.

2.2. General procedure for the Michael addition reactions

2.2.1. (*E*)-4-Acetyl-1-phenyl-3-(trifluoromethyl)hex-2-ene-1,5-dione (3aa)

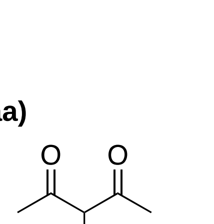

To a 30-mL two-necked round-bottomed flask containing 0.0359 g of NaH (1.50 mmol) and Et_2O (5 mL) at 0 °C under an argon atmosphere was added 0.150 mL of acetylacetone (1.46 mmol), and stirring was continued for 0.5 h where 0.192 g of 4,4,4-trifluoro-1-phenylbut-2-yn-1-one (**2a**, 0.97 mmol) was introduced, and further 0.5 h stirring was carried out. After quenching the reaction with 5 mL of 1 M aq HCl, two extractions with Et_2O afforded an organic phase that was dried with anhydrous Na_2SO_4. Concentration and purification by silica gel column chromatography using hexane/AcOEt 9:1 as an eluent furnished 0.1999 g of 4-acetyl-1-phenyl-3-(trifluoromethyl)hex-2-ene-1,5-dione (0.6702 mmol) in 69% yield as a single isomer as a yellow solid. mp 63–64 °C. Rf 0.30 (Hexane:AcOEt=8:1).

^{1}H NMR δ 2.06 (s, 6H), 7.25–7.91 (m, 6H). ^{13}C NMR δ 23.7 (q, *J*=1.2 Hz), 103.4, 123.0 (q, *J*=274.7 Hz), 128.5, 129.0, 134.0 (q, *J*=4.3 Hz), 134.3, 135.2 (q, *J*=31.6 Hz), 135.8, 189.8, 191.7. ^{19}F NMR δ −69.05 (s). IR (KBr) ν 614, 694, 783, 919, 1001, 1135, 1189, 1284, 1677, 3060 cm^{-1}. Anal. Calcd for $C_{15}H_{13}F_3O_3$, C, 60.40; H, 4.39. Found C, 60.28; H, 4.41.

2.3. General procedure for the cyclization to phenol compounds

2.3.1. 1-[2-Hydroxy-4-phenyl-6-(trifluoromethyl)phenyl]ethanone (4aa)

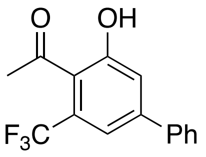

To a 30-mL two-necked round-bottomed flask containing 0.1770 g of *t*-BuOK (1.577 mmol) and Et_2O (5 mL) at 0 °C under an argon atmosphere was added 0.150 mL of acetylacetone (1.46 mmol), and stirring was continued for 0.5 h where 0.206 g of 4,4,4-trifluoro-1-phenylbut-2-yn-1-one (**2a**, 1.04 mmol) was introduced, and further 0.5-h stirring was carried out. To this mixture was added 0.1672 g of *t*-BuOK (1.490 mmol), and stirring was continued for 4 h at room temperature. After quenching the reaction with 5 mL of 1 M aq HCl, two extractions with AcOEt afforded an organic phase that was dried with anhydrous Na_2SO_4. Concentration and purification by silica gel column chromatography using hexane/AcOEt 5:1 as an eluent furnished 0.2155 g of 1-[5-hydroxy-3-(trifluoromethyl)biphenyl-4-yl]ethanone (0.7690 mmol) in 74% yield as a white solid. mp 130–131 °C. Rf 0.16 (Hexane:AcOEt=4:1). ^{1}H NMR δ 2.68 (q, *J*=1.8 Hz, 3H), 7.26–7.63 (m, 7H), 10.13 (s, 1H). ^{13}C NMR δ 31.6 (q, *J*=4.4 Hz), 117.4 (q, *J*=5.6 Hz), 119.5, 121.6 (q, *J*=1.3 Hz), 123.7 (q, *J*=273.5 Hz), 127.0, 128.88, 128.94 (q, *J*=31.6 Hz), 129.0, 138.2, 145.5, 157.5, 205.1. ^{19}F NMR δ −56.78 (s). IR (KBr) ν 761, 877, 943, 1343, 1416, 1614, 1672, 2920, 3036, 3251 cm^{-1}. Anal. Calcd for $C_{15}H_{11}F_3O_2$, C, 64.29; H, 3.96;. Found C, 63.94; H, 4.00.

2.3.2. Ethyl 2-hydroxy-4-phenyl-6-(trifluoromethyl)phenyl-1-carboxylate (4ab)

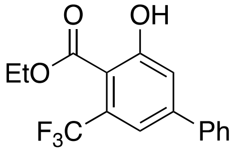

The title compound was obtained in 80% yield as a yellow oil. Rf 0.46 (Hexane: AcOEt=4:1). ^{1}H NMR δ 1.44 (t, *J*=7.1 Hz, 3H), 4.47 (q, *J*=7.2 Hz, 2H), 7.42–7.64 (m, 7H), 11.02 (s, 1H). ^{13}C NMR δ 13.5, 62.6, 109.8 (q, *J*=1.3 Hz), 118.0 (q, *J*=7.0 Hz), 119.7, 123.4 (q, *J*=272.9 Hz), 127.1, 129.0, 129.1, 130.7 (q, *J*=31.6 Hz), 138.3, 146.5, 162.4, 169.1. ^{19}F NMR δ −59.41 (s). IR (neat) ν 2987, 1672, 1620, 1298, 1143, 1060, 1015, 949, 879, 765 cm^{-1}. Anal. Calcd for $C_{16}H_{13}F_3O_3$, C, 61.94; H, 4.22. Found C, 62.11; H, 4.00.

2.3.3. 1-[2-Hydroxy-4-(4-methoxyphenyl)-6-(trifluoromethyl)phenyl]ethanone (4ba)

The title compound was obtained in 67% yield as a white solid. mp 107–108 °C. Rf 0.24 (Hexane:AcOEt=4:1). ^{1}H NMR δ 2.67 (s, 3H), 2.68 (q, *J*=1.9 Hz, 3H), 7.30 (d, *J*=7.8 Hz, 2H), 7.39

(d, *J*=1.5 Hz, 1H), 7.49 (d, *J*=1.8 Hz, 1H), 7.52 (d, *J*=8.1 Hz, 2H), 10.25 (s, 1H). ^{13}C NMR δ 31.5 (q, *J*=4.4 Hz), 55.3, 114.4, 116.8 (q, *J*=5.6 Hz), 118.6, 120.9, 123.7 (q, *J*=271.5 Hz), 128.1, 128.8 (q, *J*=23.9 Hz), 130.4, 145.0, 157.7, 160.3, 205.1. ^{19}F NMR δ –56.69 (s). IR (KBr) ν 3204, 2843, 1686, 1608, 1526, 1340, 1262, 1136, 944, 831 cm^{-1}. Anal. Calcd for $C_{18}H_{13}F_3O_3$, C, 61.94; H, 4.22. Found C, 61.82; H, 4.00.

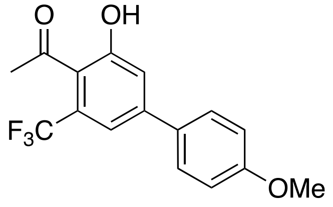

2.3.4. Ethyl 2-hydroxy-4-(4-methoxyphenyl)-6-(trifluoromethyl)phenyl-1-carboxylate (4bb)

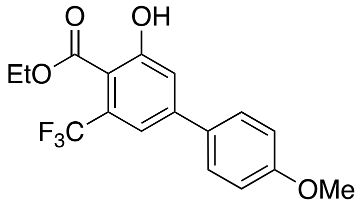

The title compound was obtained in 73% yield as a yellow solid. mp 60–61 °C. Rf 0.29 (Hexane:AcOEt=5:1). ^{1}H NMR δ 1.43 (t, *J*=7.2 Hz, 3H), 3.87 (s, 3H), 4.46 (q, *J*=7.2 Hz, 2H), 7.00 (m, 2H), 7.37 (d, *J*=1.8 Hz, 1H), 7.52 (d, *J*=1.5 Hz, 1H), 7.57 (m, 2H), 11.01 (s, 1H). ^{13}C NMR δ 13.4, 55.2, 62.4, 108.9, 114.4, 117.4 (q, *J*=7.0 Hz), 118.7, 123.4 (q, *J*=273.6 Hz), 128.2, 130.4, 130.5 (q, *J*=31.4 Hz), 145.9, 160.4, 162.4, 169.1. ^{19}F NMR δ –59.33 (s). IR (KBr) ν 2996, 1659, 1606, 1294, 1034, 947, 874, 829, 710, 600 cm^{-1}. Anal. Calcd for $C_{17}H_{15}F_3O_4$, C, 60.00; H, 4.44. Found C, 59.91; H, 4.46.

2.3.5. 1-[2-Hydroxy-4-(4-methylphenyl)-6-(trifluoromethyl)phenyl]ethanone (4ca) [6]

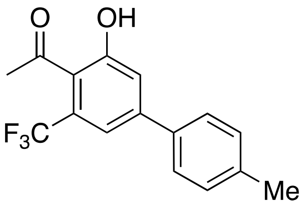

The title compound was obtained in 68% yield as a white solid. Rf 0.26 (Hexane:AcOEt =4:1). ^{1}H NMR δ 2.42 (s, 3H), 2.67 (q, *J*=1.8 Hz, 3H), 7.29 (d, *J*=8.1 Hz, 2H), 7.38 (d, *J*=1.5 Hz, 1H), 7.48–7.52 (m, 3H), 10.13 (s). ^{13}C NMR (acetone-*d*$_6$) δ 21.0, 31.8, 116.2, 118.4, 124.7 (q, *J*=273.2 Hz), 127.6, 127.9, 128.4 (q, *J*=30.8 Hz), 130.5, 136.7, 139.2, 144.2, 155.4, 201.8. ^{19}F NMR δ –56.73 (s).

2.3.6. Ethyl [2-hydroxy-4-(4-methylphenyl)-6-(trifluoromethyl)phenyl]-1-carboxylate (4cb)

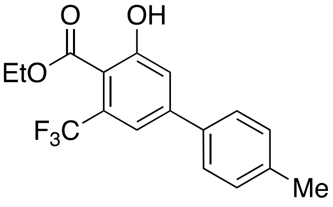

The title compound was obtained in 75% yield as a white solid. mp 64–69 °C. Rf 0.31 (Hexane:AcOEt=5:1). ^{1}H NMR δ 1.43 (t, *J*=7.2 Hz, 3H), 2.42 (s, 3H), 4.47 (q, *J*=7.2 Hz, 2H), 7.29 (d, *J*=8.4 Hz, 2H), 7.41 (d, *J*=1.8 Hz, 1H), 7.51–7.54 (m, 3H), 11.02 (s, 1H). ^{13}C NMR δ 13.4, 21.0, 62.4, 109.3, 117.7 (q, *J*=6.8 Hz), 119.2, 123.4 (q, *J*=271.3 Hz), 126.8, 129.7, 130.5 (q, *J*=31.4 Hz), 135.2, 139.1, 146.3, 162.4, 169.1. ^{19}F NMR δ –59.25 (s). IR (KBr) ν 2988, 1663, 1020, 949, 882, 821, 712, 660, 559, 497 cm^{-1}. Anal. Calcd for $C_{17}H_{15}F_3O_3$, C, 62.96; H, 4.66. Found C, 62.89; H, 4.47.

2.3.7. 1-[2-Hydroxy-4-(4-bromophenyl)-6-(trifluoromethyl)phenyl]ethanone (4da) [6]

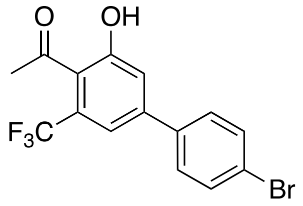

The title compound was obtained in 70% yield as a white solid. mp 136 °C. Rf 0.17 (Hexane:AcOEt=5:1). ^{1}H NMR δ 2.68 (qd, *J*=1.8, 0.9 Hz, 3H), 7.35 (m, 1H), 7.44–7.49 (m, 3H), 7.59–7.64 (m, 2H), 9.94 (s, 1H). ^{13}C NMR δ 31.6 (q, *J*=4.9 Hz), 117.2 (q, *J*=5.6 Hz), 119.6, 121.0, 123.5, 123.6 (q, *J*=271.9 Hz), 128.6, 129.4 (q, *J*=31.4 Hz), 132.3, 137.0, 144.5, 158.3, 204.8. ^{19}F NMR δ –56.82 (s). IR (KBr) ν 3193, 2925, 1690, 1613, 1342, 1136, 1012, 941, 878 826 cm^{-1}. Anal. Calcd for $C_{15}H_{10}F_3O_2$, C, 50.16; H, 2.81. Found C, 50.11; H, 2.48.

2.3.8. Ethyl [4-(4-bromophenyl)-2-hydroxy-6-(trifluoromethyl)]phenyl-1-carboxylate (4db)

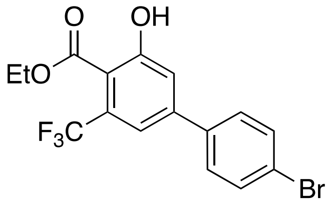

The title compound was obtained in 54% yield as a white solid. mp 93–94 °C. Rf 0.40 (Hexane:AcOEt=5:1). ^{1}H NMR δ 1.44 (t, *J*=7.2 Hz, 3H), 4.47 (q, *J*=7.2 Hz, 2H), 7.38 (d, *J*=1.8 Hz, 1H), 7.46–7.50 (m, 3H), 7.60–7.64 (m, 2H), 11.01 (s, 1H). ^{13}C NMR δ 13.4, 62.6, 110.0, 117.5 (q, *J*=6.8 Hz), 119.5, 123.2 (q, *J*=271.3 Hz), 123.5, 128.5, 130.8 (q, *J*=31.7 Hz), 132.2, 137.0, 145.1, 162.3, 168.9. ^{19}F NMR δ –59.40 (s). IR (KBr) ν 3087, 2986, 1671, 1330, 1059, 1011, 949, 828, 720, 567 cm^{-1}. Anal. Calcd for $C_{16}H_{12}F_3O_3$, C, 49.38; H, 3.11. Found C, 49.41; H, 2.76.

2.4. 3-Phenyl-5-(trifluoromethyl)phenol (5a) [7]

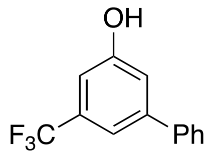

To a 30-mL two-necked round-bottomed flask containing 0.0556 g of *t*-BuOK (0.495 mmol) and *t*-BuOH (3 mL) at 0 °C under an argon atmosphere was added a solution of 0.1525 g of 4-acetyl-1-phenyl-3-trifluoromethylhex-2-ene-1,5-dione (0.5113 mmol) in 2 mL of *t*-BuOH, and stirring was continued for 4 h at 60 °C. After quenching the reaction with 3 mL of 1 M aq HCl aq., two extractions with AcOEt afforded an organic phase that was dried with anhydrous Na_2SO_4. Concentration and purification by silica gel column chromatography using hexane/AcOEt 5:1 as an eluent furnished 0.0981 g of 3-phenyl-5-(trifluoromethyl)phenol (0.4118 mmol) in 81% yield as a yellow oil. Rf 0.34 (Hexane:AcOEt=4:1). ^{1}H NMR δ 7.07 (brs, 1H), 7.23 (brs, 1H), 7.37–7.50 (m, 4H), 7.56–7.59 (m, 2H). ^{13}C NMR δ 111.0 (q, *J*=3.7 Hz), 116.5 (q, *J*=3.9 Hz), 117.3, 123.8 (q, *J*=271.7 Hz), 127.1, 128.2, 128.9, 132.4 (q, *J*=31.4 Hz), 139.4, 143.8, 156.0. ^{19}F NMR δ –63.69 (s).

2.5. General procedure for the preparation of the trifluoromethylated pyrimidines

2.5.1. 2-Amino-4-phenyl-6-(trifluoromethyl)pyrimidine (6aa) [8].

To a 30-mL two-necked round-bottomed flask containing 0.1295 g of Na_2CO_3 (1.222 mmol)

and CH_3CN (5 mL), 0.0574 g of guanidine hydrochloride (0.601 mmol) was added. After heating to 80 °C, 0.1029 g of 4,4,4-trifluoro-1-phenylbut-2-yn-1-one (**2a**, 0.501 mmol) was added, and the mixture was stirred for 8 h at that temperature. After quenching the reaction with 3 mL of 1 M aq HCl aq., two extractions with AcOEt afforded an organic phase that was dried with anhydrous Na_2SO_4. Concentration and purification by silica gel column chromatography using hexane/AcOEt 10:1 as an eluent and the following recrystallization furnished 0.0723 g of 2-amino-4-phenyl-6-(trifluoromethyl)pyrimidine (0.3023 mmol) in 60% yield as a white solid. mp 128–129 °C. Rf 0.11 (Hexane:AcOEt=10:1). ^{1}H NMR δ 5.67 (brs, 2H), 7.33 (s, 1H), 7.50-7.52 (m, 3H), 8.03 (dd, *J*=7.8, 1.8 Hz, 2H). ^{13}C NMR (DMSO-d_6) δ 100.9 (q, *J*=2.8 Hz), 121.0 (q, *J*=274.8 Hz), 127.2, 128.8, 131.4, 135.9, 156.1 (q, *J*=33.9 Hz), 164.0, 167.0. ^{19}F NMR δ –71.91 (s).

2.5.2. 2-Amino-4-(4-methoxyphenyl)-6-(trifluoromethyl)pyrimidine (6ab) [9]

The title compound was obtained in 55% yield as a white solid. mp 191–192 °C. Rf 0.05 (Hexane:AcOEt=10:1). ^{1}H NMR δ 3.89 (s, 3H), 5.38 (brs, 2H), 7.00 (d, *J*=9.0 Hz, 2H), 7.29 (s, 1H), 8.03 (d, *J*=9.0 Hz, 2H). ^{13}C NMR (DMSO-d_6) δ 55.4, 100.1 (d, *J*=2.5 Hz), 114.2, 121.1 (q, *J*=275.0 Hz), 128.2, 129.0, 155.8 (q, *J*=33.7 Hz), 162.0, 163.8, 166.4. ^{19}F NMR δ –71.98 (s).

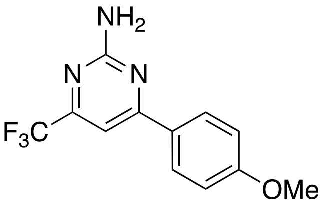

2.5.3. 2-Amino-4-(4-methylphenyl)-6-(trifluoromethyl)pyrimidine (6ac) [10]

The title compound was obtained in 52% yield as a yellow solid. mp 174–175 °C. Rf 0.11 (Hexane:AcOEt=10:1). ^{1}H NMR δ 2.43 (s, 3H), 5.33 (brs, 2H), 7.31 (d, *J*=8.1 Hz, 2H), 7.33 (s, 1H), 7.95 (d, *J*=8.1 Hz, 2H). ^{13}C NMR (DMSO-d_6) δ 21.0, 100.5, 121.1 (q, *J*=275.1 Hz), 127.2, 129.5, 133.2, 141.5, 156.0 (q, *J*=33.9 Hz), 164.0, 166.9. ^{19}F NMR δ –71.98 (s).

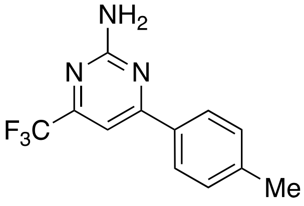

2.5.4. 2-Amino-4-(4-bromophenyl)-6-(trifluoromethyl)pyrimidine (6ad) [10]

The title compound was obtained in 67% yield as a brownish solid. mp 215–216 °C. Rf 0.20 (Hexane:AcOEt=10:1). ^{1}H NMR δ 5.41 (br s, 2H), 7.32 (s, 1H), 7.65 (d, *J*=8.7 Hz, 2H), 7.93 (d, *J*=8.4 Hz, 2H). ^{13}C NMR (DMSO-d_6) δ 100.8 (d, *J*=2.5 Hz), 120.9 (q, *J*=273.8 Hz), 125.3, 129.2, 131.9, 135.1, 156.3 (q, *J*=34.1 Hz), 163.9, 165.8. ^{19}F NMR δ –71.98 (s). IR (KBr) *ν* 3486, 3318, 3205, 1643, 1593, 1556, 1481, 1382, 1281, 1259, 1182, 1164, 1140, 1070, 1011, 822 cm^{-1}. HRMS-FAB (m/z) : [M+H]$^+$ calcd. for $C_{11}H_8BrF_3N_3$, 317.9848; found, 317.9820.

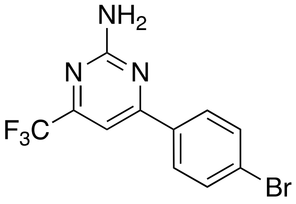

2.5.5. 2-Methyl-4-phenyl-6-(trifluoromethyl)pyrimidine (6ba) [8]

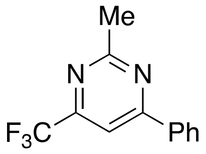

The title compound was obtained in 67% yield as a yellow oil. Rf 0.43 (Hexane:AcOEt= 10:1). ^{1}H NMR δ 2.89 (s, 3H), 7.51-7.56 (m, 3H), 7.83 (s, 1H), 8.12-8.15 (m, 2H). ^{13}C NMR δ 26.1, 109.4 (q, *J*=2.9 Hz), 120.7 (q, *J*=274.8 Hz), 127.4, 129.1, 131.7, 135.7, 156.1 (q, *J*=35.4 Hz), 166.5, 169.5. ^{19}F NMR δ −71.34 (s).

2.5.6. 4-(4-Methoxyphenyl)-2-methyl-6-(trifluoromethyl)pyrimidine (6bb) [11]

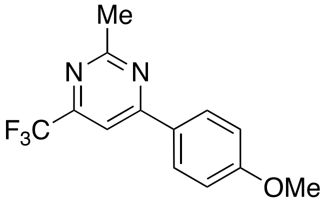

The title compound was obtained in 75% yield as a yellow solid. mp 82–83 °C. Rf 0.28 (Hexane:AcOEt=10:1). ^{1}H NMR δ 2.85 (s, 3H), 3.90 (s, 3H), 7.01-7.06 (m, 2H), 7.76 (s, 1H), 8.10-8.15 (m, 2H). ^{13}C NMR δ 26.1, 55.4, 108.4 (q, *J*=2.9 Hz), 114.4, 120.8 (q, *J*=274.6 Hz), 128.0, 129.0, 155.8 (q, *J*=35.2 Hz), 162.7, 165.8, 169.3. ^{19}F NMR δ −71.41 (s). IR (KBr) *v* 3076, 2979, 2945, 2845, 1549, 1389, 1312, 1298, 1266, 1183, 1144, 1027, 840 cm^{-1}. HRMS-FAB (m/z): $[M+2H]^{+2}$ calcd. for $C_{13}H_{13}F_3N_2O$, 270.0980; found, 270.0994.

2.5.7. 2-Methyl-4-(4-methylphenyl)-6-(trifluoromethyl)pyrimidine (6bc) [12]

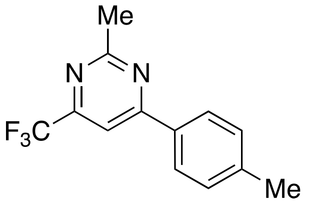

The title compound was obtained in 53% yield as a yellow solid. mp 64–65 °C. Rf 0.42 (Hexane:AcOEt=10:1). ^{1}H NMR δ 2.44 (s, 3H), 2.87 (s, 3H), 7.34 (d, *J*=8.1 Hz, 2H), 7.80 (s, 1H), 8.04 (d, *J*=8.1 Hz, 2H). ^{13}C NMR δ 21.4, 26.1, 109.0 (q, *J*=2.7 Hz), 120.8 (q, *J*=274.6 Hz), 127.3, 129.8, 132.9, 142.4, 156.0 (q, *J*=35.1 Hz), 166.3, 169.4. ^{19}F NMR δ −71.37 (s). IR (KBr) *v* 3036, 2928, 1592, 1550, 1389, 1286, 1264, 1190, 1134, 877, 838 cm^{-1}. HRMS-FAB (m/z) : $[M+H]^{+}$ calcd. for $C_{13}H_{11}F_3N_2$, 252.0874; found, 252.0845.

2.5.8. 4-(4-Bromophenyl)-2-methyl-6-(trifluoromethyl)-pyrimidine (6bd) [11]

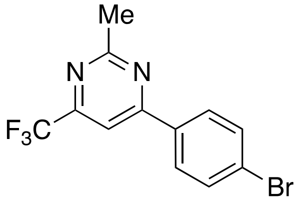

The title compound was obtained in 57% yield as a white solid. mp 79–80 °C. Rf 0.45 (Hexane:AcOEt=10:1). ^{1}H NMR δ 2.89 (s, 3H), 7.68 (d, *J*=8.7 Hz, 2H), 7.81 (s, 1H), 8.03 (d, *J*=8.7 Hz, 2H). ^{13}C NMR δ 26.1, 109.1 (q, *J*=2.9 Hz), 120.6 (q, *J*=275.0 Hz), 126.7, 128.8, 132.3, 134.5, 156.4 (q, *J*=35.6 Hz), 165.2, 169.6. ^{19}F NMR δ −71.36 (s). IR (KBr) *v* 3087, 3009, 2975, 2932, 1592, 1548, 1394, 1274, 1257, 1203, 1158, 1072, 1009, 833 cm^{-1}. HRMS-FAB (m/z) : $[M+H]^{+}$ calcd. for $C_{12}H_9BrF_3N_2$, 316.9896; found, 316.9857.

2.5.9. 4-Phenyl-6-(trifluoromethyl)pyrimidine (6ca) [8]

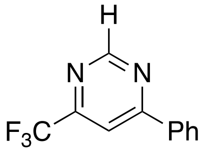

The title compound was obtained in 21% yield as a yellow solid. mp 41–42 °C. Rf 0.42 (Hexane:AcOEt=10:1). ^{1}H NMR δ 7.53-7.60 (m, 3H), 8.05 (s, 1H), 8.16 (dd, *J*=7.7, 2.0 Hz, 2H), 9.41 (s, 1H). ^{13}C NMR δ 112.5 (q, *J*=2.9 Hz), 120.6 (q, *J*=274.8 Hz), 127.4, 129.2, 132.1, 135.3, 156.1 (q, *J*=35.8 Hz), 159.4, 166.5. ^{19}F NMR δ −71.31 (s).

2.5.10. 4-(4-Methoxyphenyl)-6-(trifluoromethyl)pyrimidine (6cb) [11]

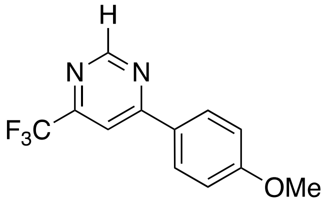

The title compound was obtained in 28% yield as a white solid. mp 52–53 °C. Rf 0.24 (Hexane:AcOEt=10:1). ^{1}H NMR δ 3.91 (s, 3H), 7.04 (d, *J*=8.7 Hz, 2H), 7.95 (s, 1H), 8.14 (d, *J*=9.0 Hz, 2H), 9.31 (s, 1H). ^{13}C NMR δ 55.4, 111.4 (q, *J*=2.7 Hz), 114.6, 120.7 (q, *J*=274.4 Hz), 127.6, 129.1, 155.7 (q, *J*=35.8 Hz), 159.2, 163.0, 165.8. ^{19}F NMR δ −71.39 (s). IR (KBr) *v* 3079, 2972, 2924, 2847, 1596, 1539, 1518, 1394, 1309, 1270, 1175, 1139, 1095, 1063, 1022, 840 cm^{-1}. Anal. Calcd for $C_{12}H_9F_3N_2O$, C, 56.70; H, 3.57; N, 11.02. Found C, 56.67; H, 3.47; N, 10.79.

2.5.11. 4-(4-Methylphenyl)-6-(trifluoromethyl)pyrimidine (6cc)

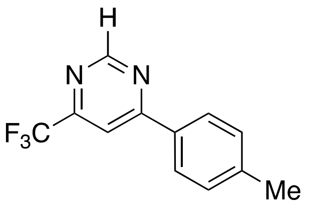

The title compound was obtained in 22% yield as a white solid. mp 53–54 °C. Rf 0.49 (Hexane:AcOEt=10:1). ^{1}H NMR δ 2.45 (s, 3H), 7.35 (d, *J*=8.1 Hz, 2H), 8.00 (s, 1H), 8.06 (d, *J*= 7.5 Hz, 2H), 9.36 (s, 1H). ^{13}C NMR δ 21.5, 112.1 (d, *J*=2.5 Hz), 120.7 (q, *J*=274.6 Hz), 127.3, 130.0, 132.5, 142.9, 155.9 (q, *J*=35.7 Hz), 159.3, 166.4. ^{19}F NMR δ −71.34 (s). IR (KBr) *v* 3033, 2925, 1597, 1541, 1392, 1322, 1303, 1255, 1144, 1096, 1060, 836, 822 cm^{-1}. Anal. Calcd for $C_{12}H_9F_3N_2$, C, 60.51; H, 3.81; N, 11.76. Found C, 60.51; H, 3.84; N, 11.73.

2.5.12. 4-(4-Bromophenyl)-6-(trifluoromethyl)pyrimidine (6cd)

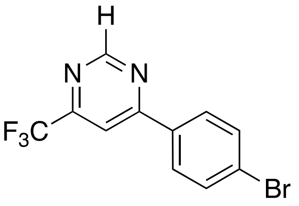

The title compound was obtained in 26% yield as a yellow solid. mp 39–40 °C. Rf 0.37 (Hexane:AcOEt=10:1). ^{1}H NMR δ 7.70 (d, *J*=8.4 Hz, 2H), 8.02 (d, *J*=6.0 Hz, 2H), 8.06 (s, 1H), 9.40 (s, 1H). ^{13}C NMR δ 112.3 (q, *J*=2.5 Hz), 120.5 (q, *J*=274.6 Hz), 127.1, 128.8, 132.5, 134.1, 156.4 (q, *J*=35.4 Hz), 159.5, 165.3. ^{19}F NMR δ −71.32 (s). IR (KBr) *v* 3070, 1595, 1537, 1403, 1324, 1307, 1290, 1264, 1202, 1133, 1095, 835 cm^{-1}. Anal. Calcd for $C_{11}H_6BrF_3N_2$, C, 43.59; H, 2.00; N, 9.24. Found C, 43.55; H, 1.68; N, 9.10.

3. Spectral data

4,4,4-Trifluoro-1-phenylbut-2-yn-1-one (2a)

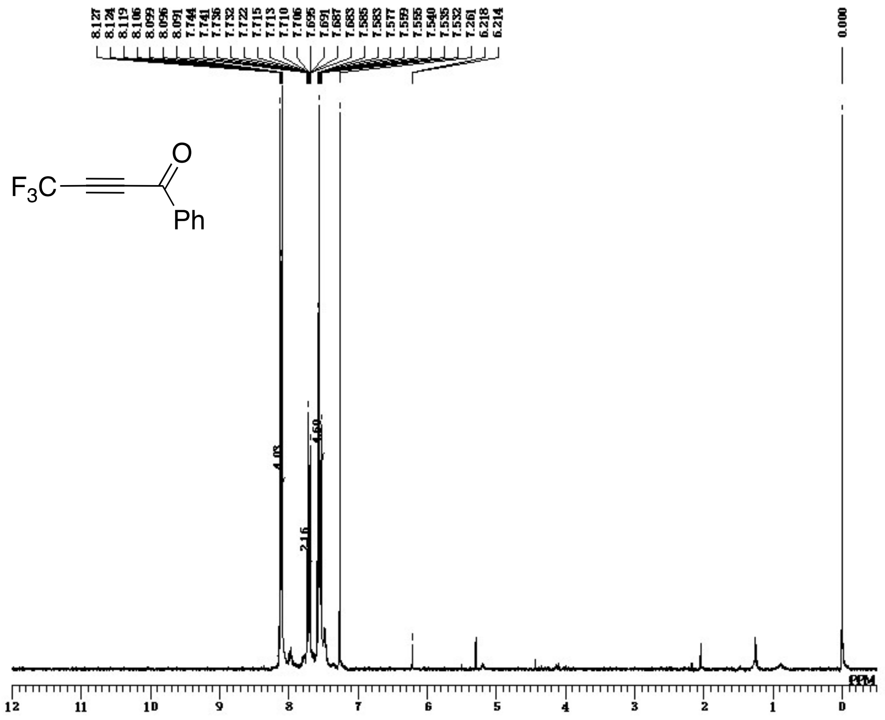

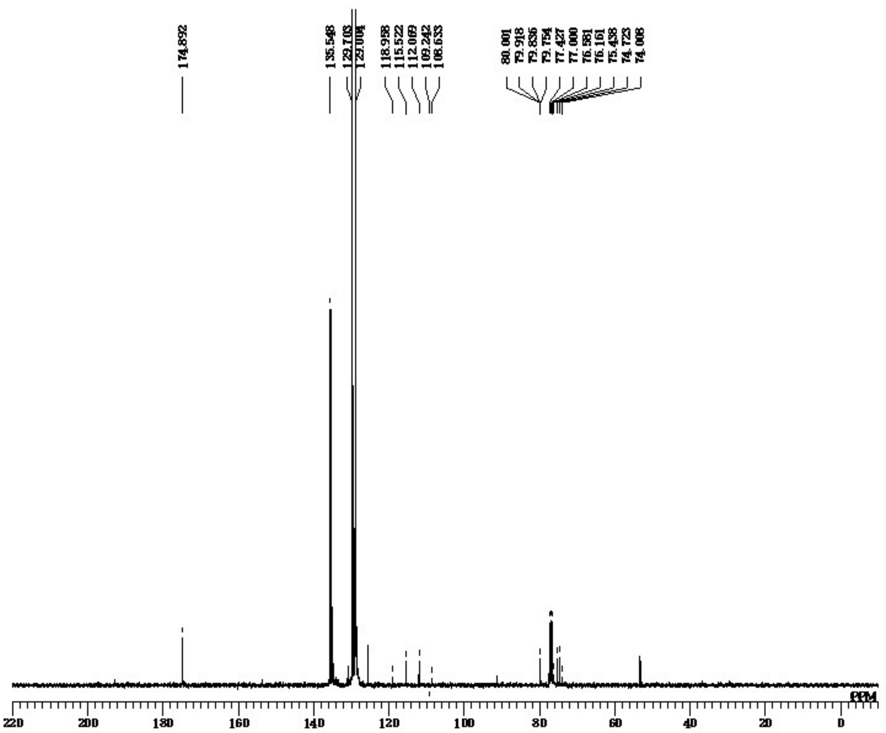

4,4,4-Trifluoro-1-(4-methoxyphenyl)but-2-yn-1-one (2b)

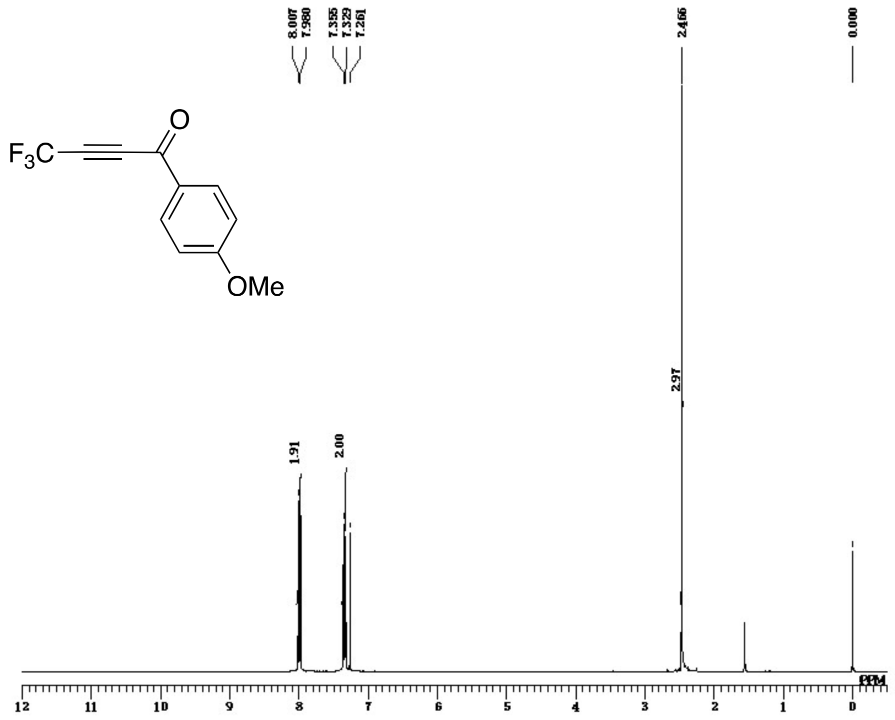

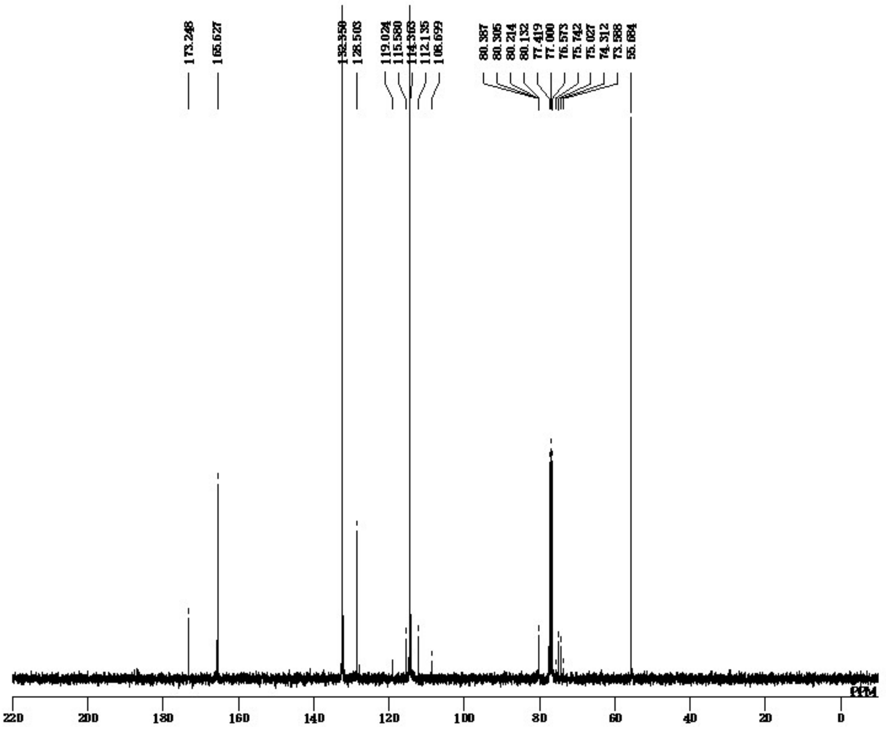

4,4,4-Trifluoro-1-(4-methylphenyl)but-2-yn-1-one (2c)

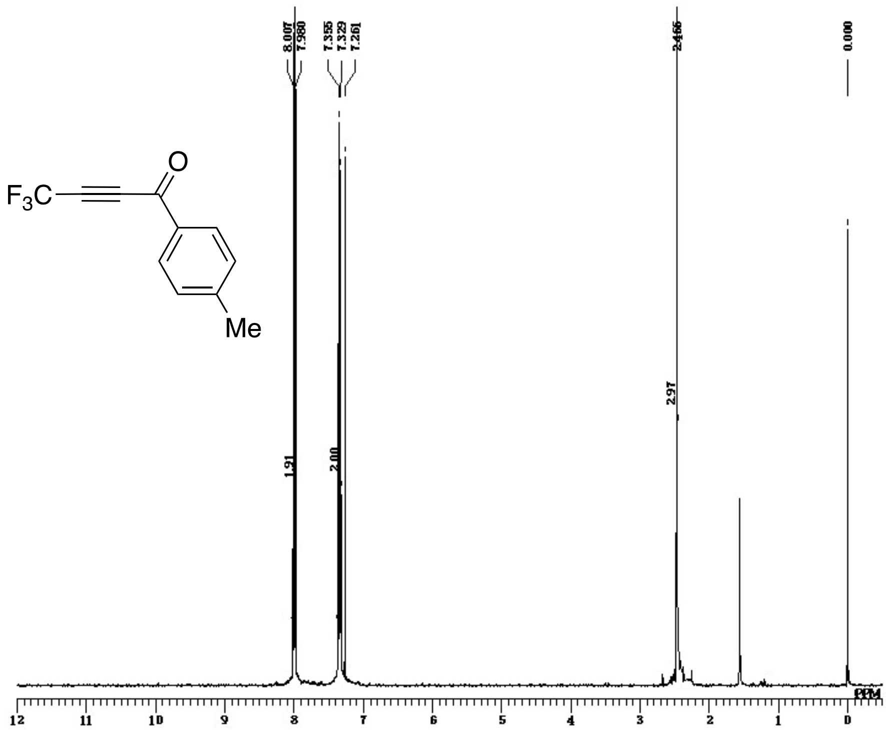

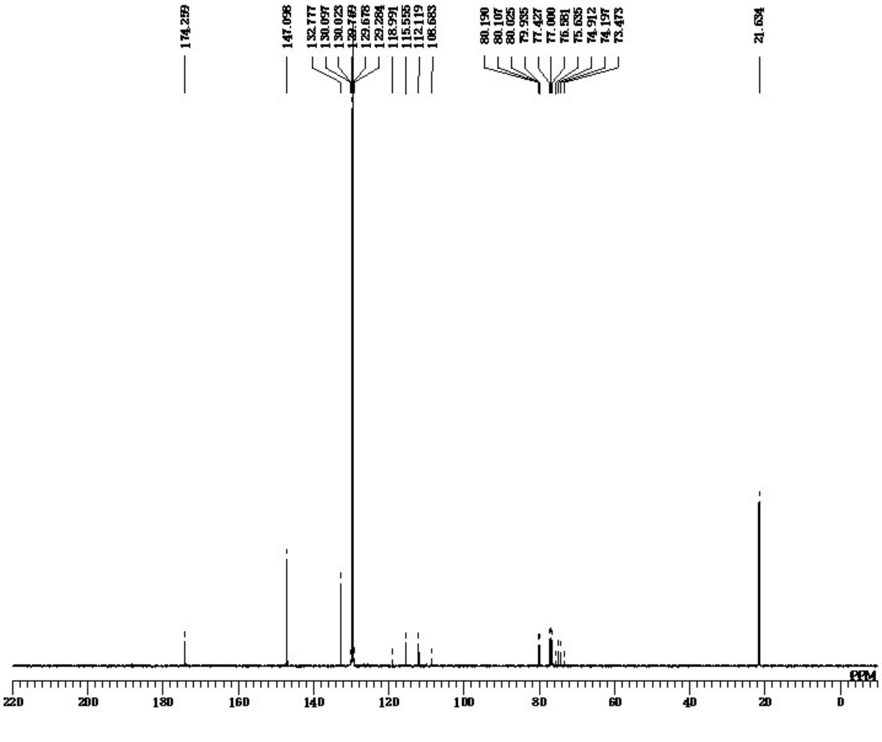

1-(4-Bromophenyl)-4,4,4-trifluorobut-2-yn-1-one (2d)

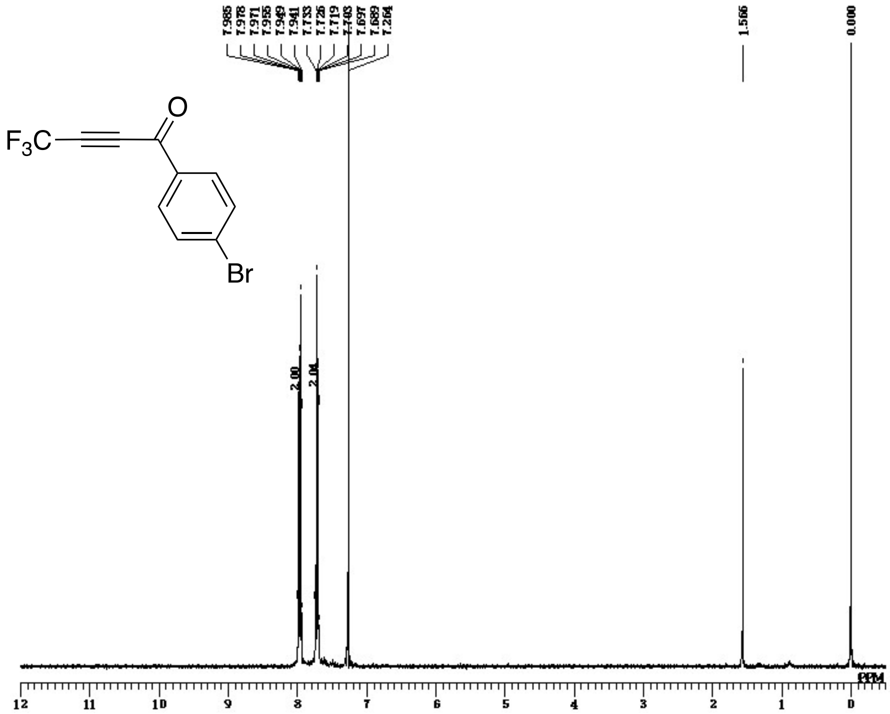

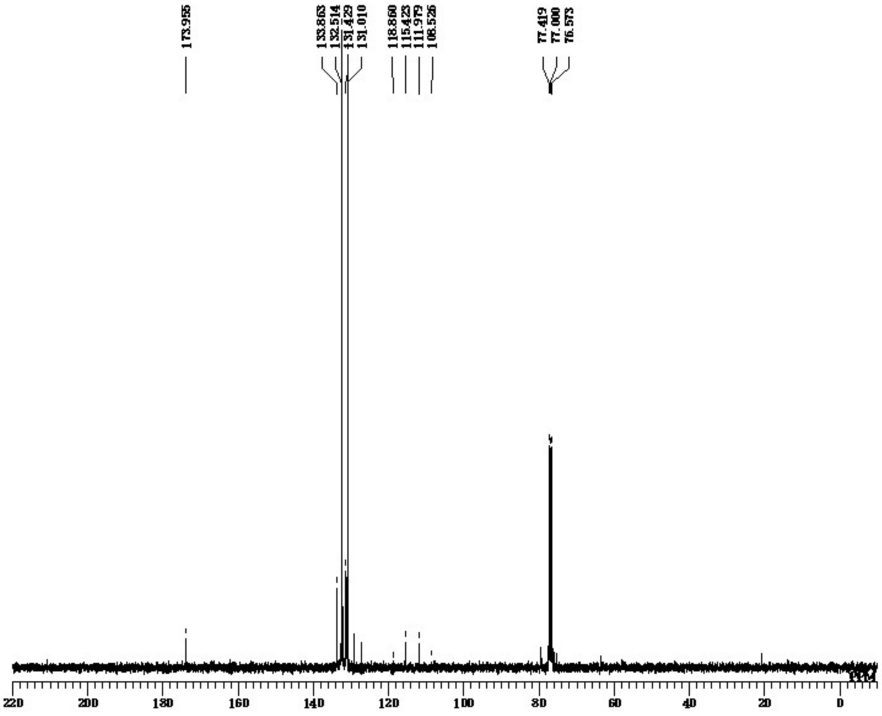

(*E*)-4-Acetyl-1-phenyl-3-(trifluoromethyl)hex-2-ene-1,5-dione (3aa)

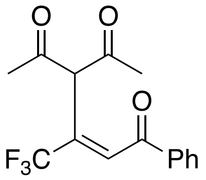

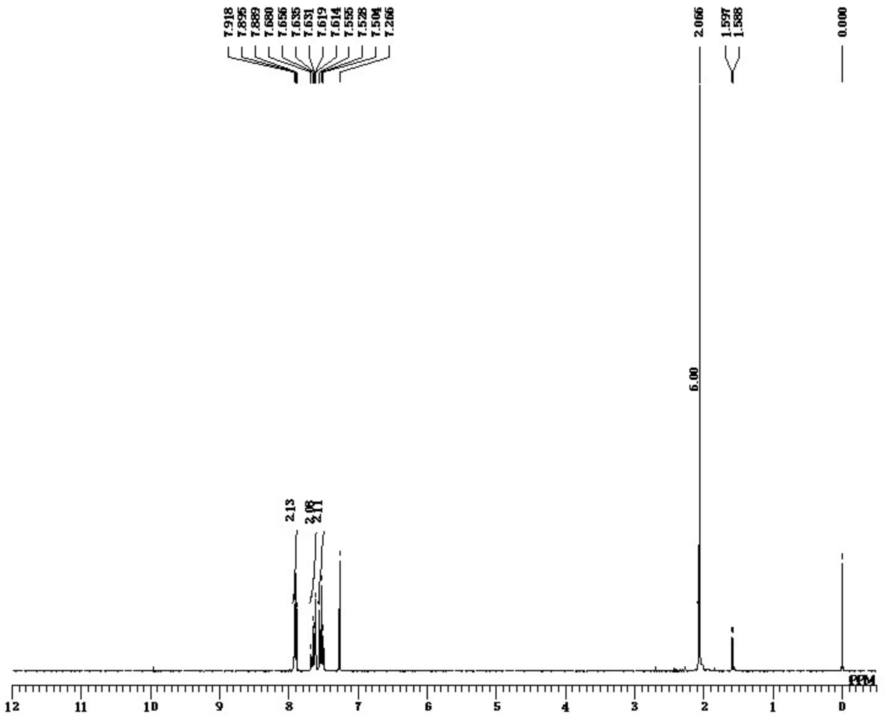

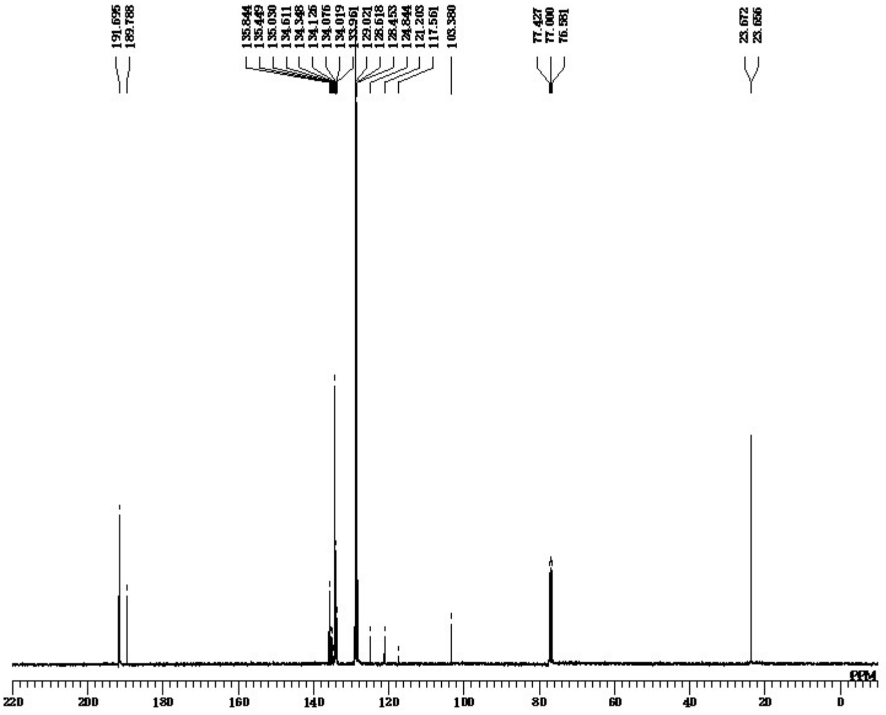

1-[2-Hydroxy-4-phenyl-6-(trifluoromethyl)phenyl]ethanone (4aa)

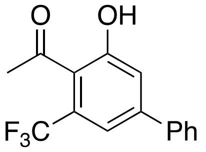

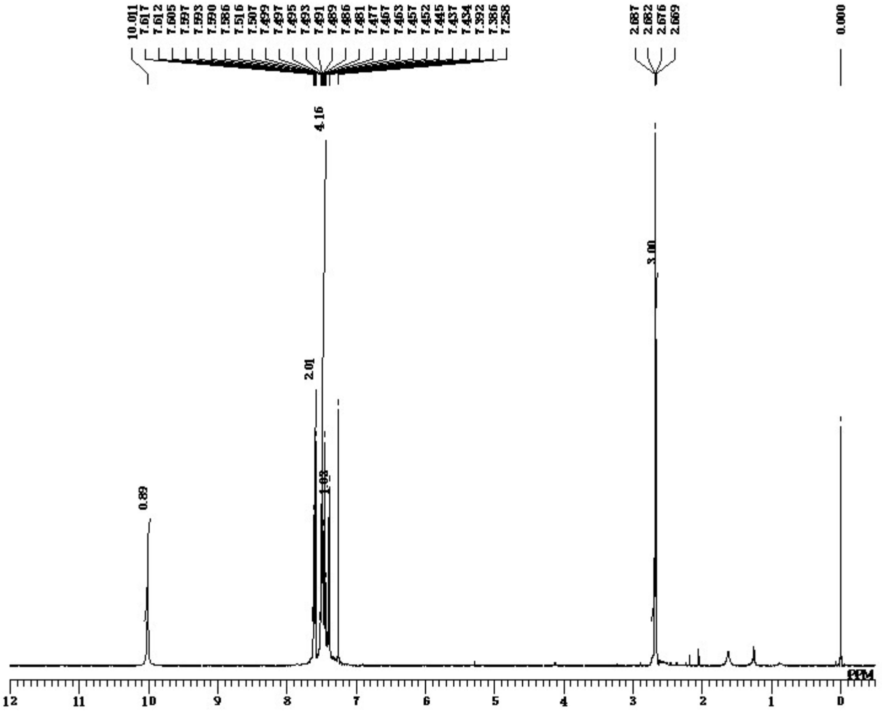

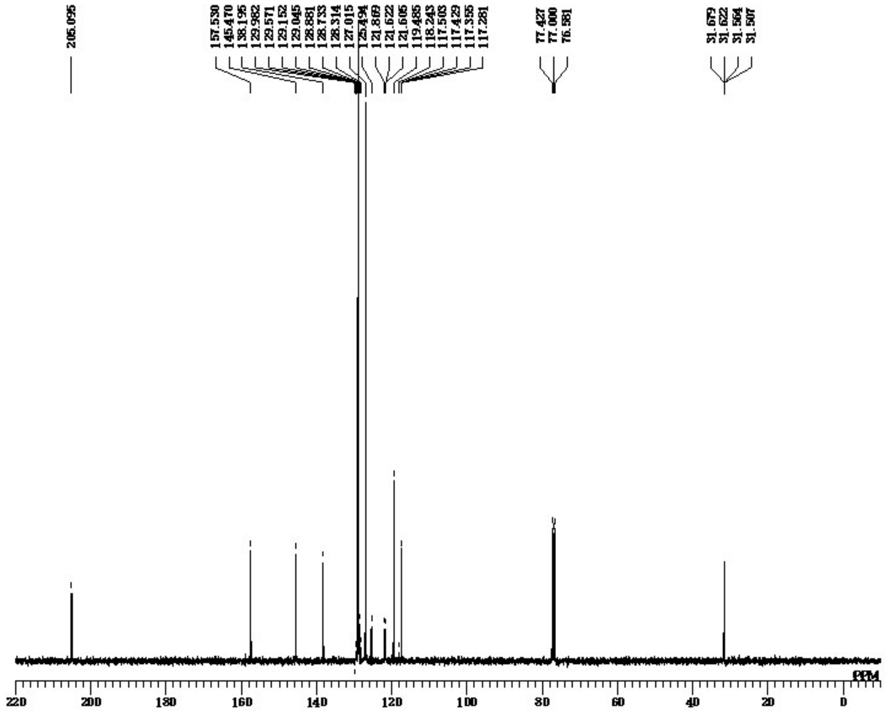

Ethyl 2-hydroxy-4-phenyl-6-(trifluoromethyl)phenyl-1-carboxylate (4ab)

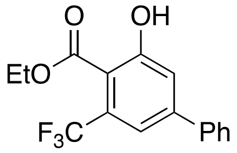

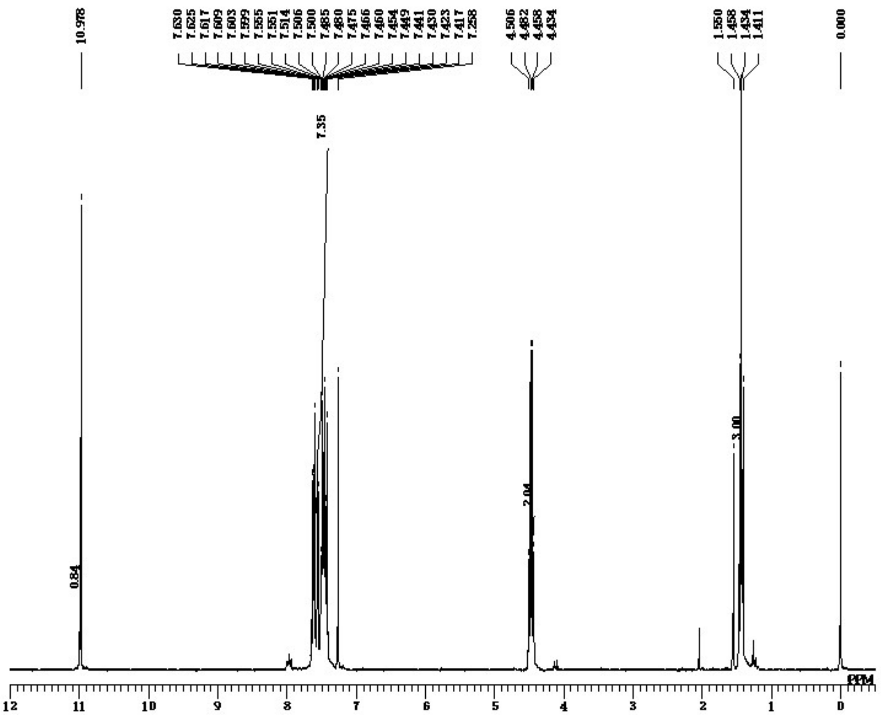

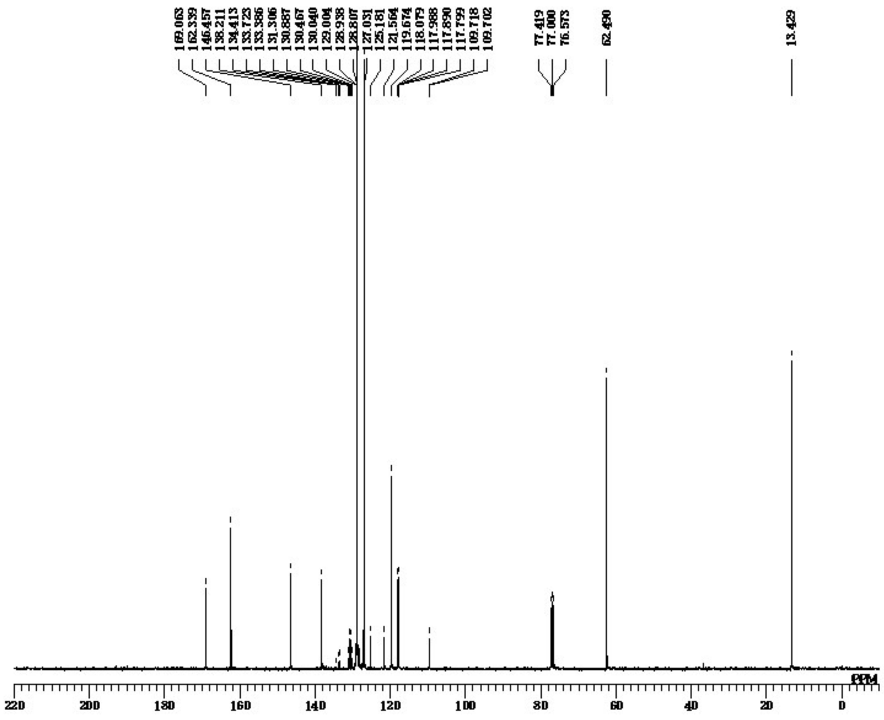

1-[2-Hydroxy-4-(4-methoxyphenyl)-6-(trifluoromethyl)phenyl]ethanone (4ba)

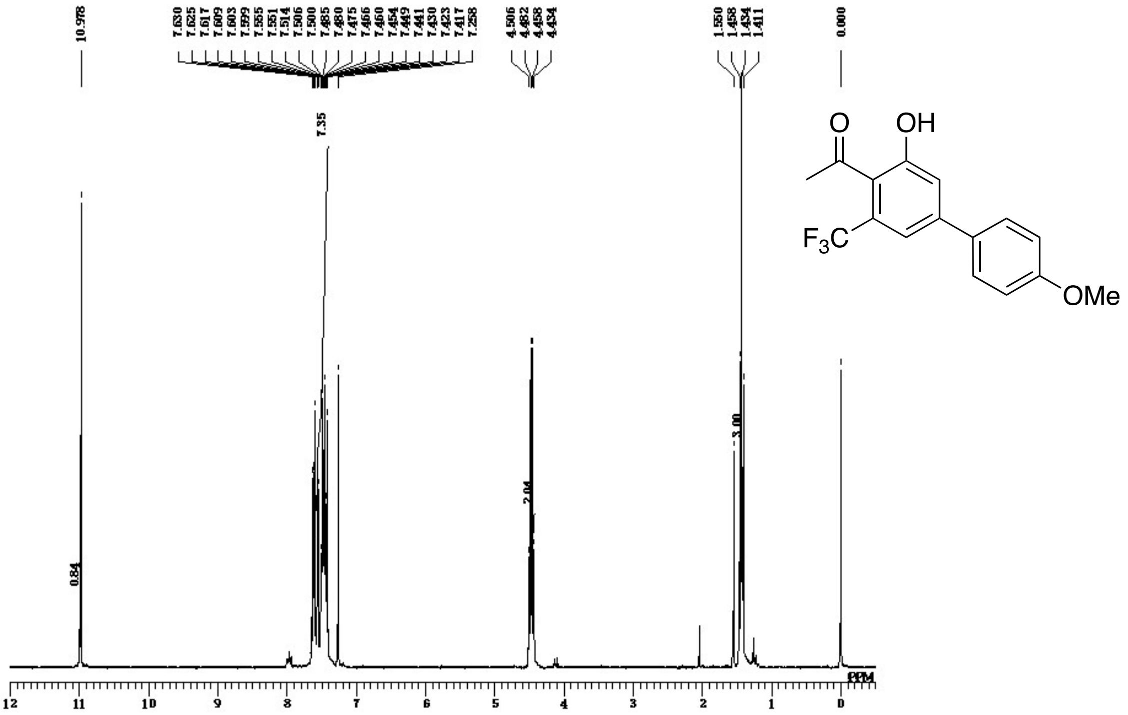

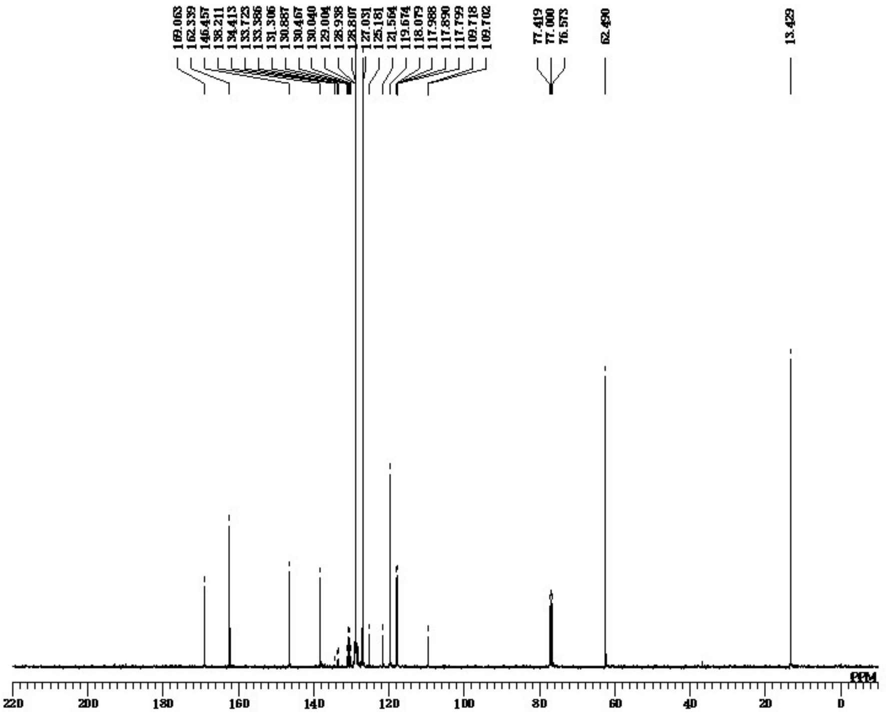

Ethyl 2-hydroxy-4-(4-methoxyphenyl)-6-(trifluoromethyl)phenyl-1-carboxylate (4bb)

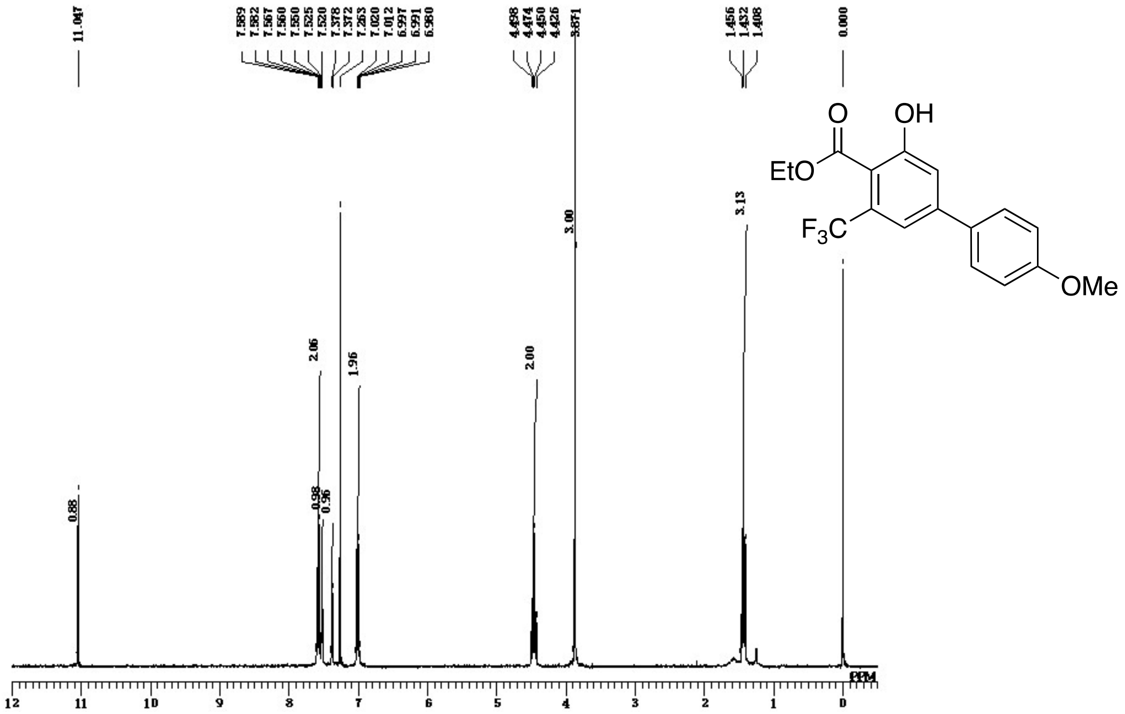

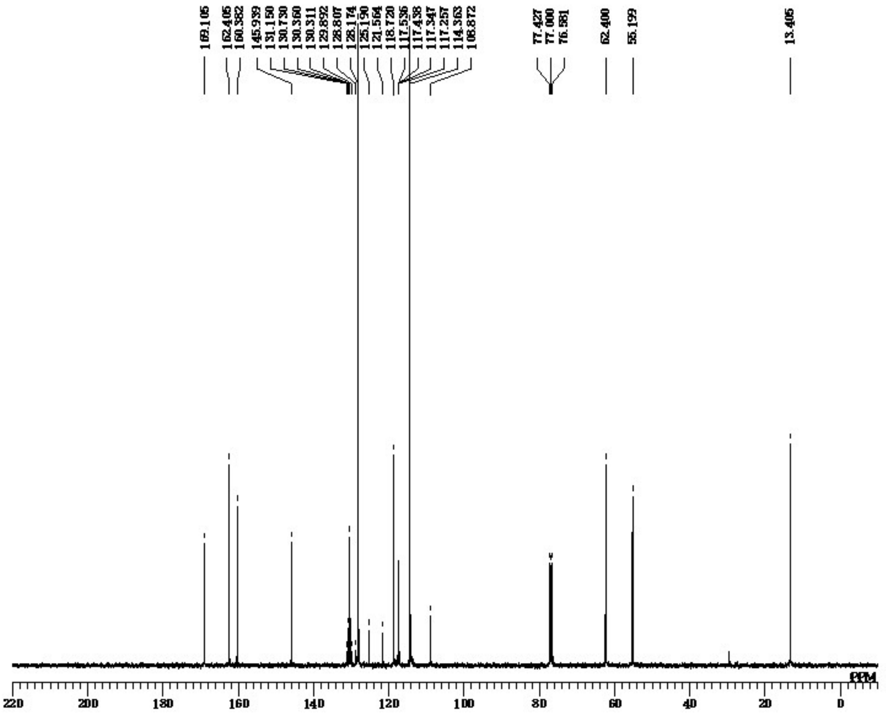

1-[2-Hydroxy-4-(4-methylphenyl)-6-(trifluoromethyl)phenyl]ethanone (4ca)

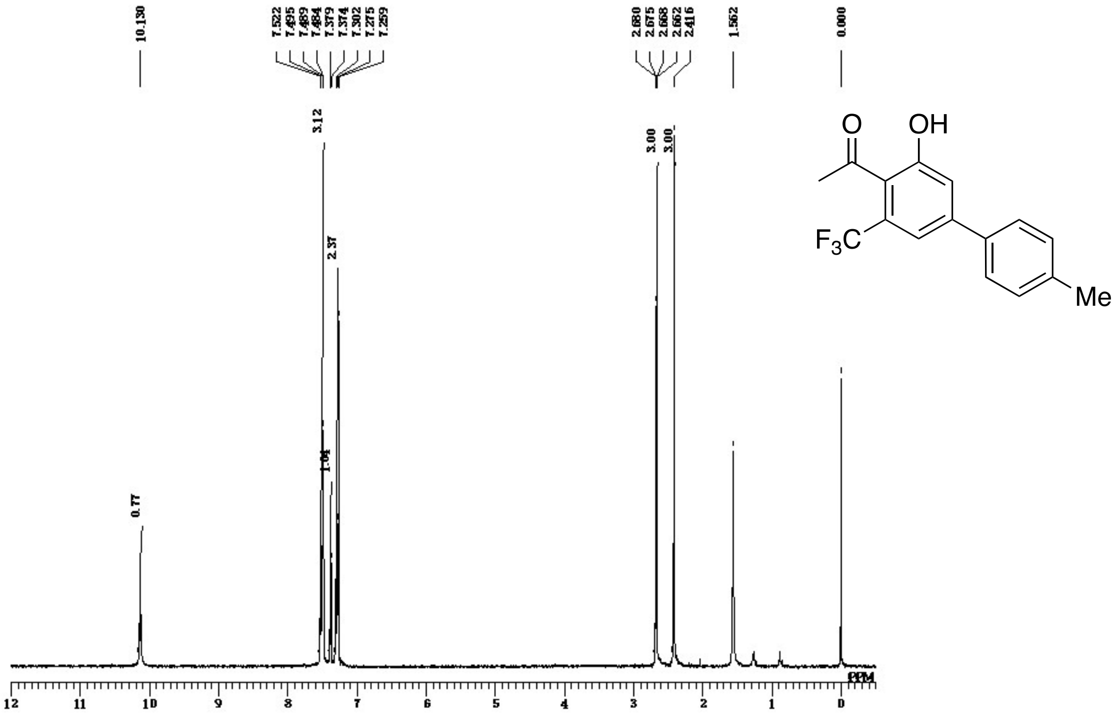

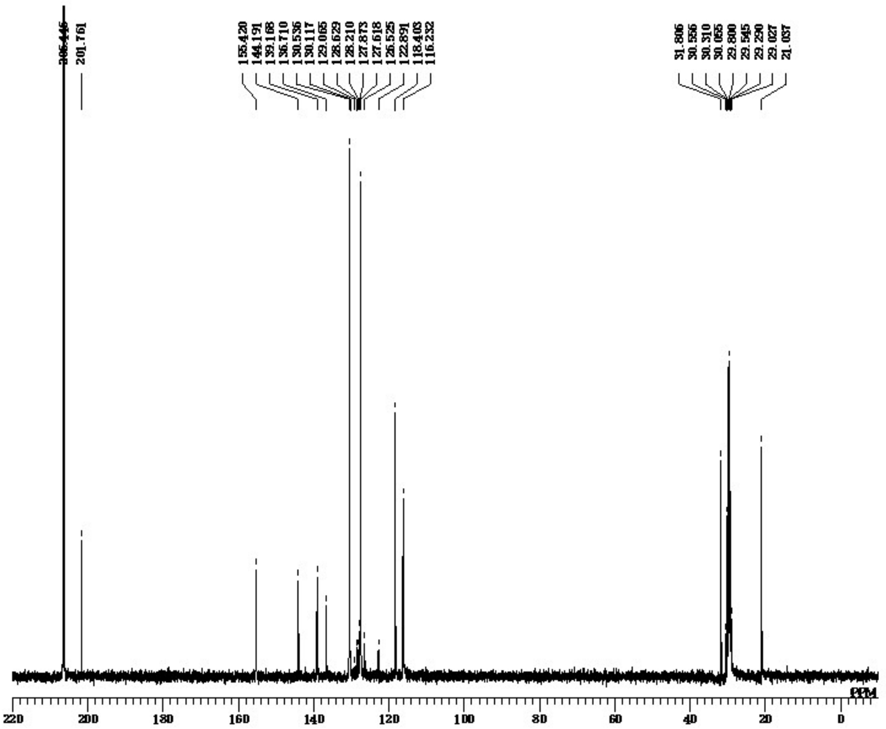

Ethyl [2-hydroxy-4-(4-methylphenyl)-6-(trifluoromethyl)phenyl]-1-carboxylate (4cb)

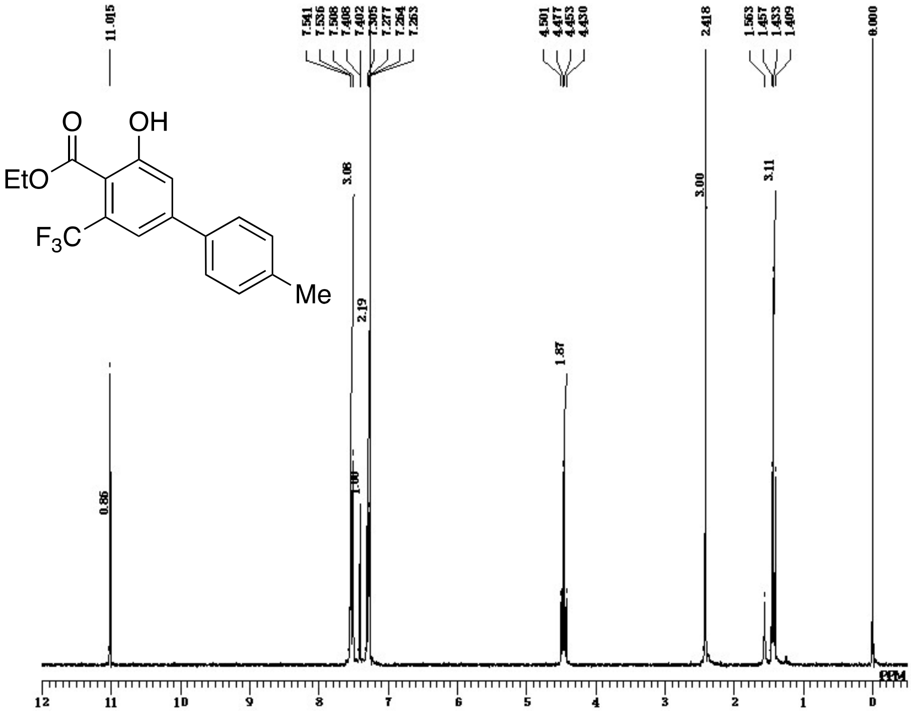

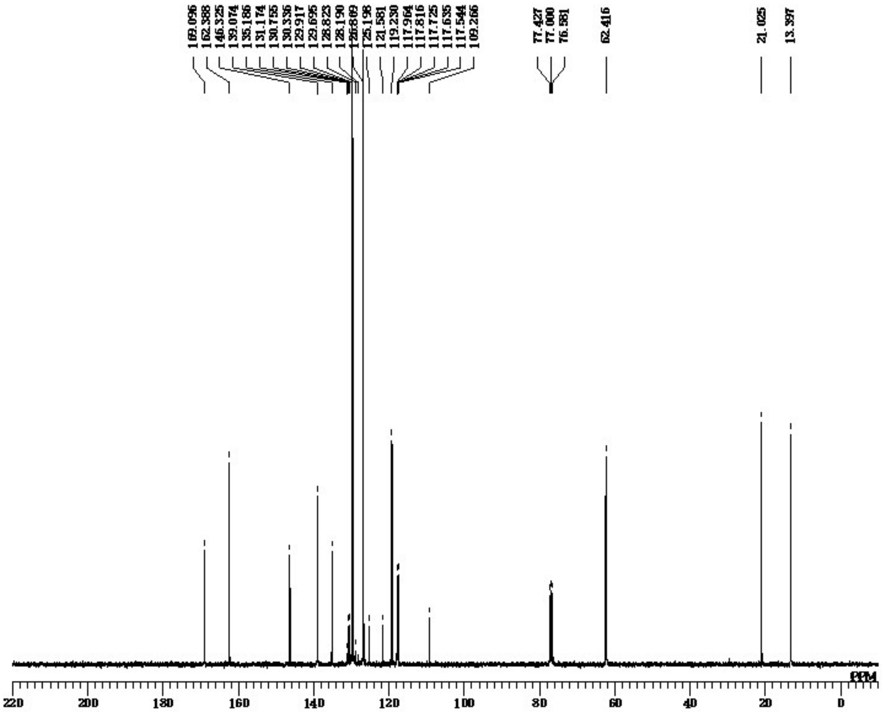

2.4.7. 1-[2-Hydroxy-4-(4-bromophenyl)-6-(trifluoromethyl)phenyl]ethanone (4da)

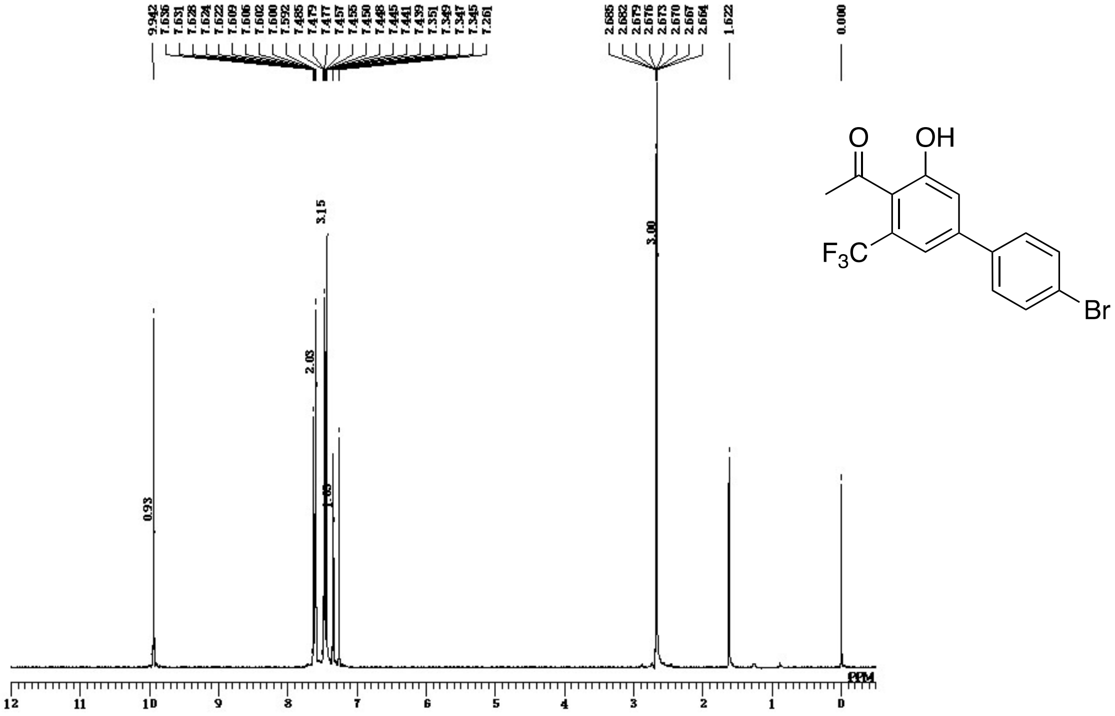

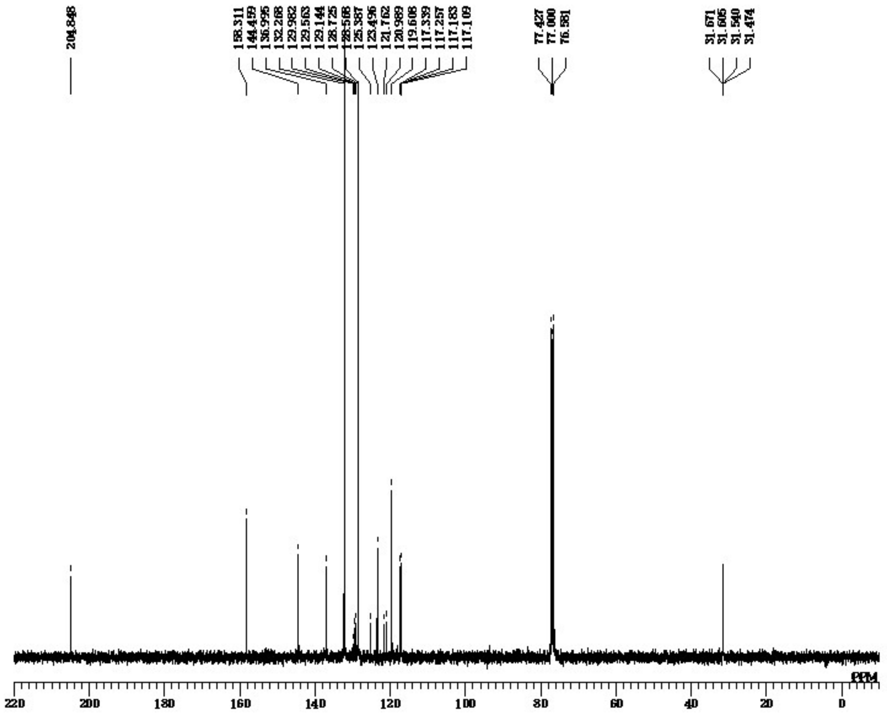

Ethyl [4-(4-bromophenyl)-2-hydroxy-6-(trifluoro-methyl)]phenyl-1-carboxylate (4db)

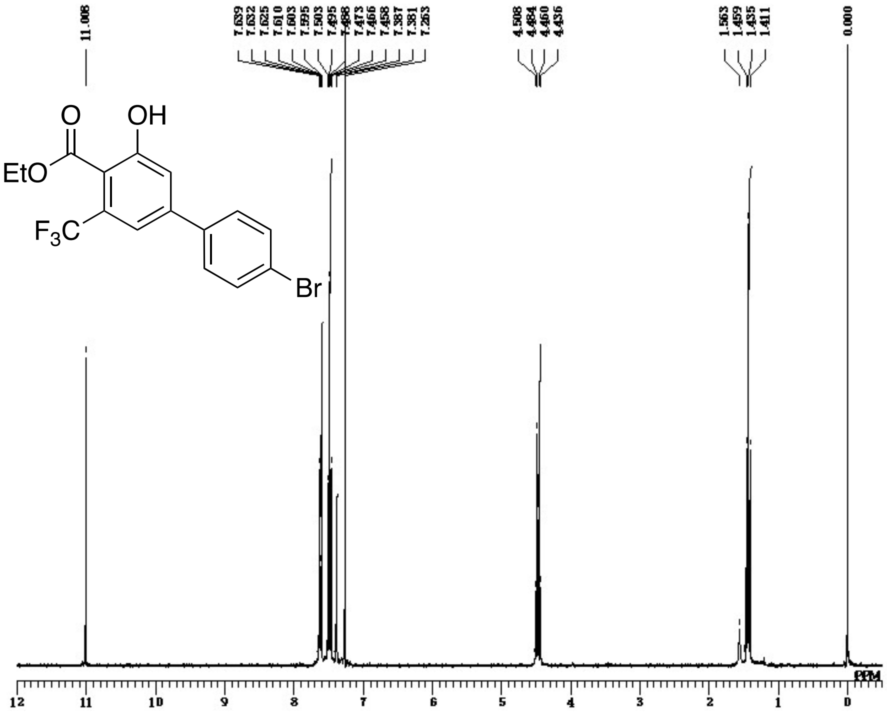

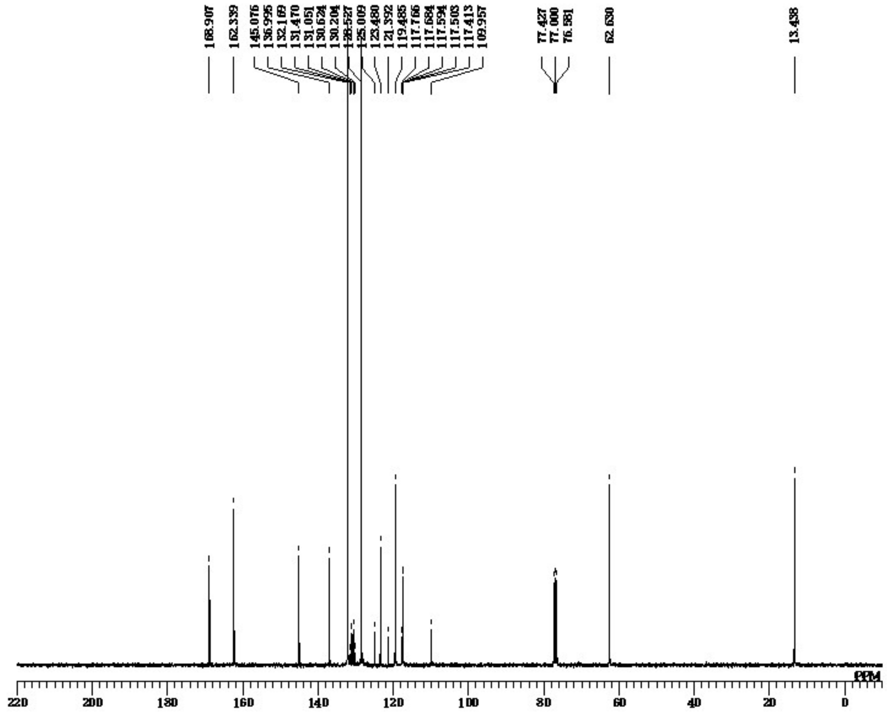

3-Phenyl-5-(trifluoromethyl)phenol (5a)

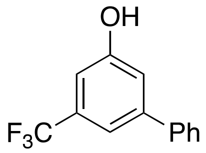

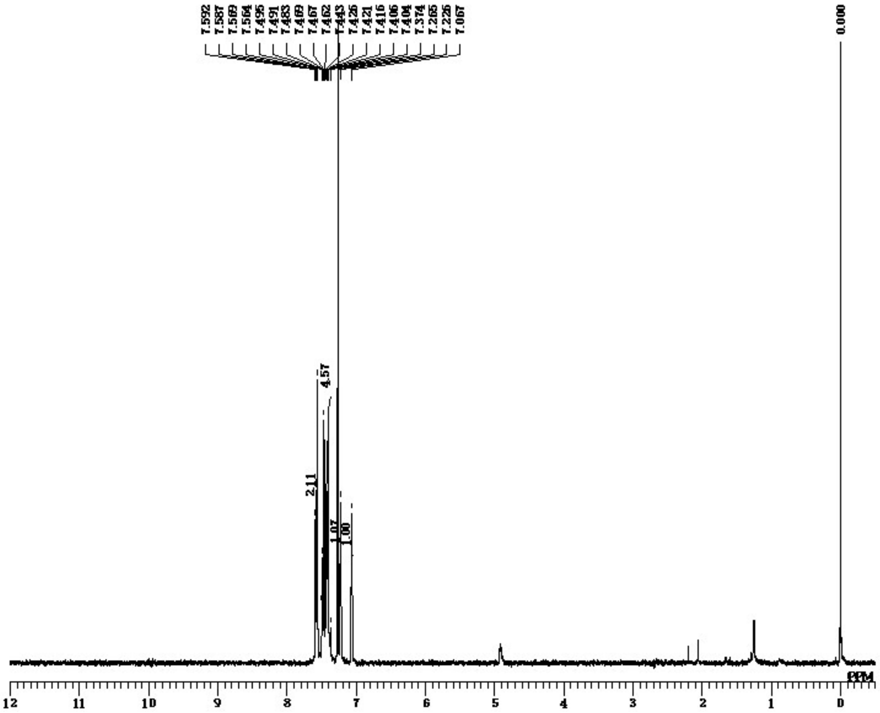

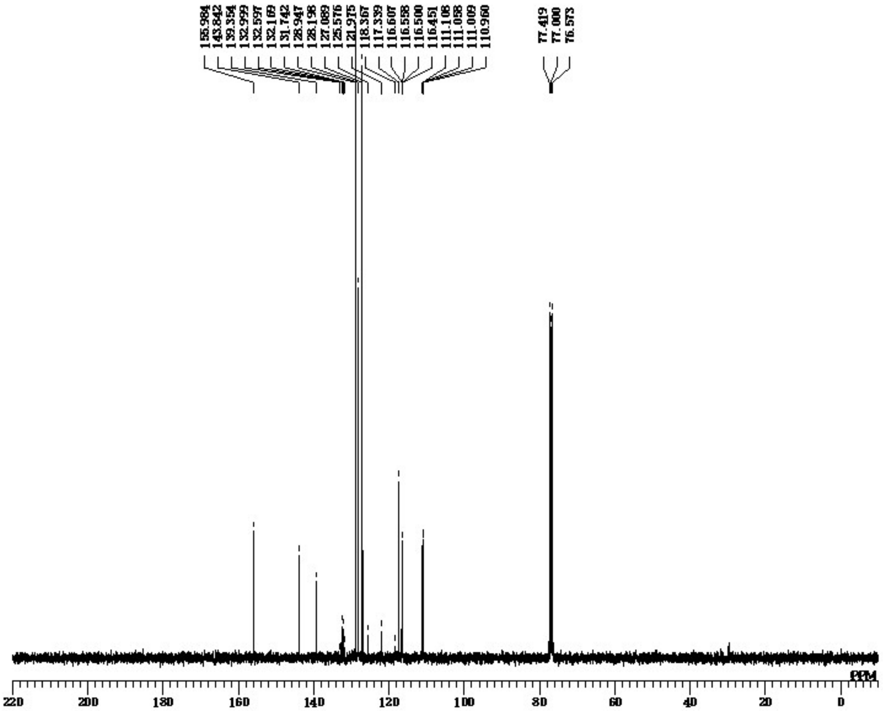

2-Amino-4-phenyl-6-(trifluoromethyl)pyrimidine (6aa)

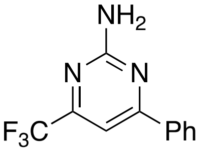

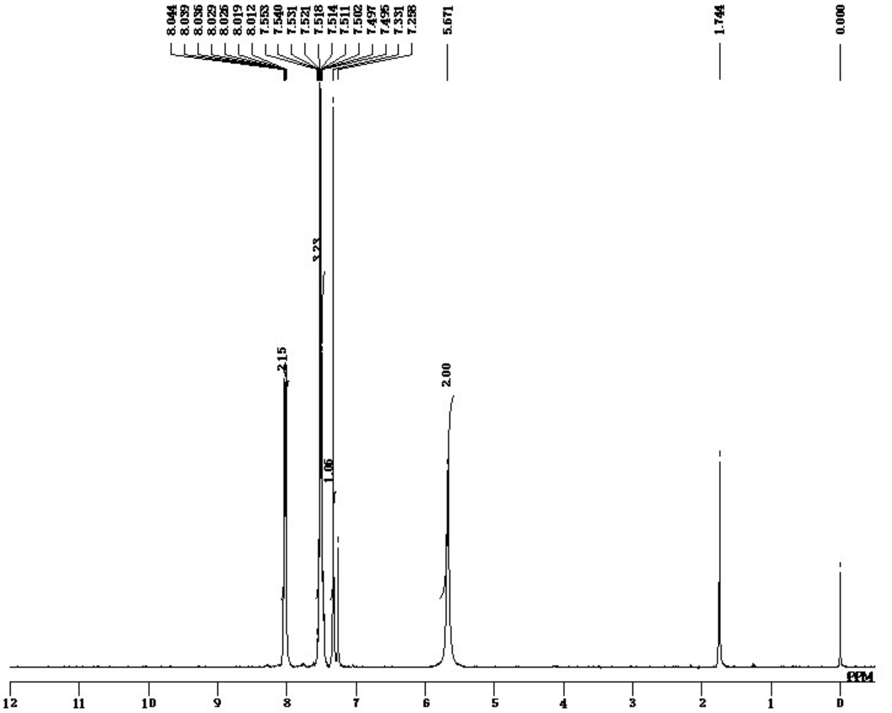

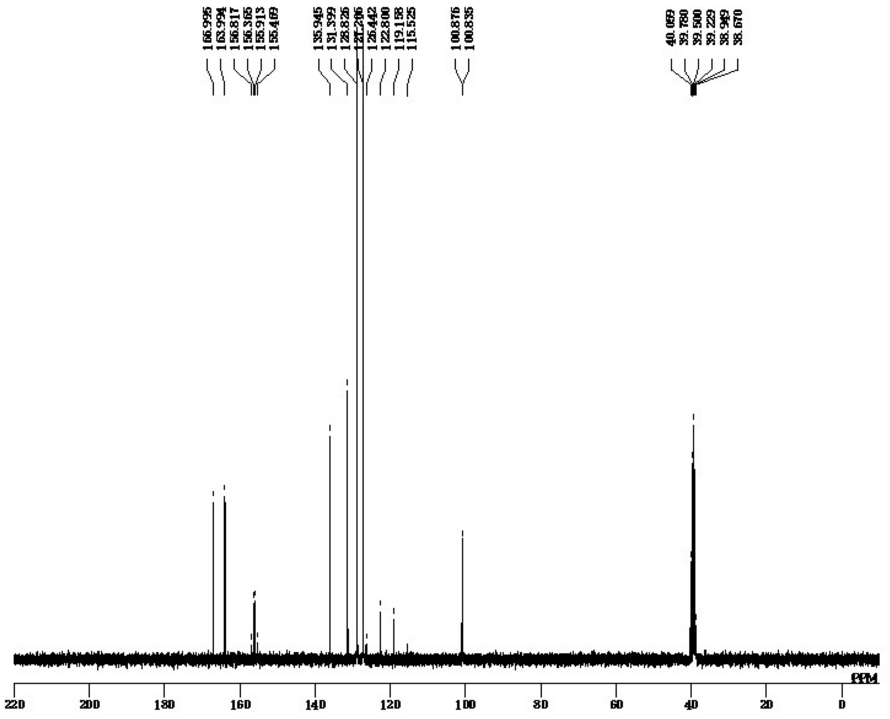

2-Amino-4-(4-methoxyphenyl)-6-(trifluoromethyl)pyrimidine (6ab)

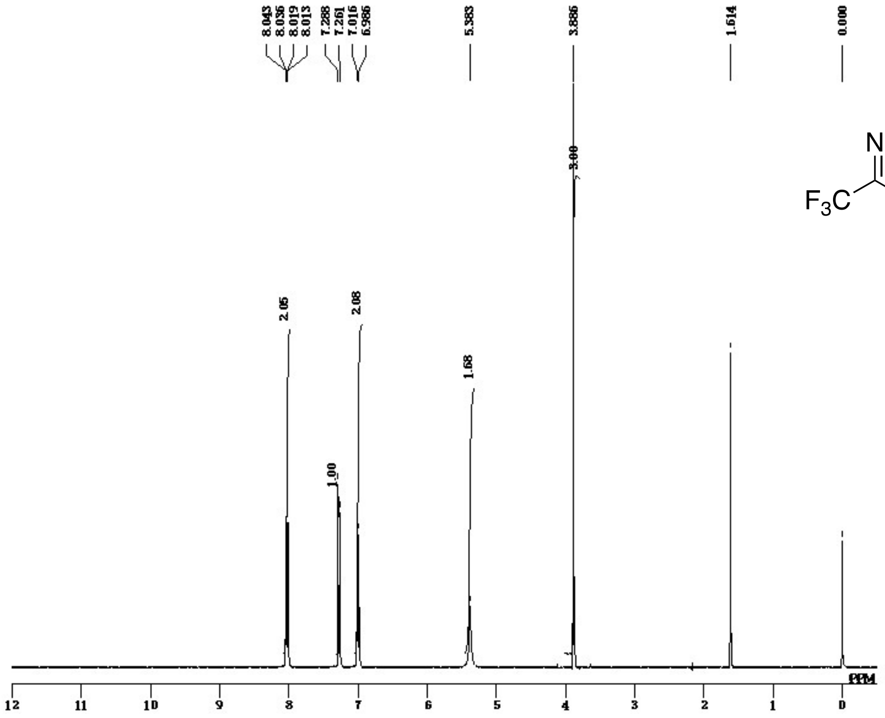

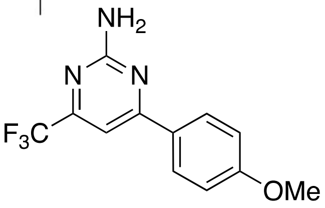

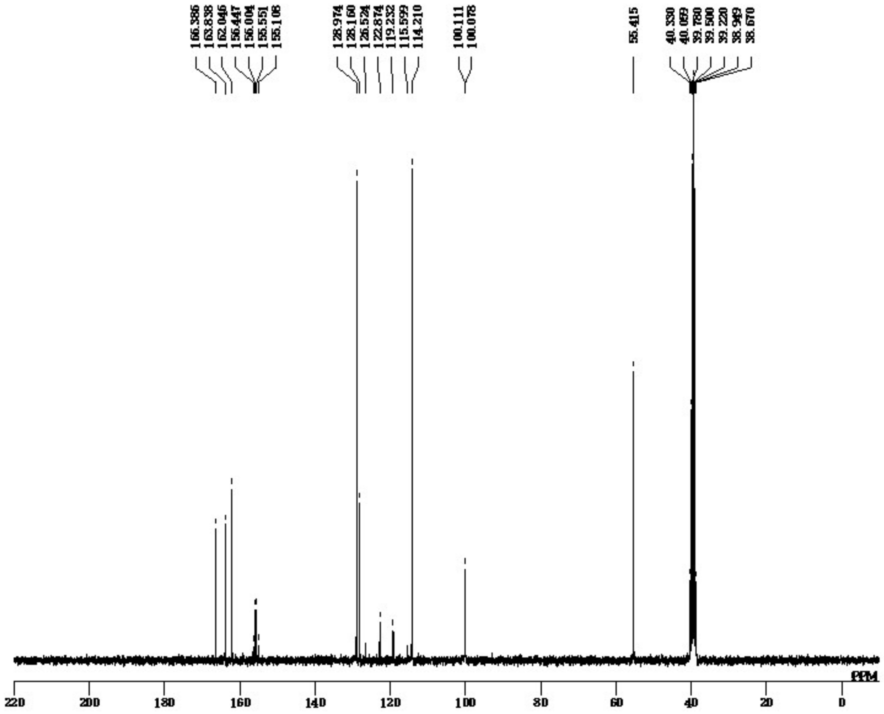

2-Amino-4-(4-methylphenyl)-6-(trifluoromethyl)pyrimidine (6ac)

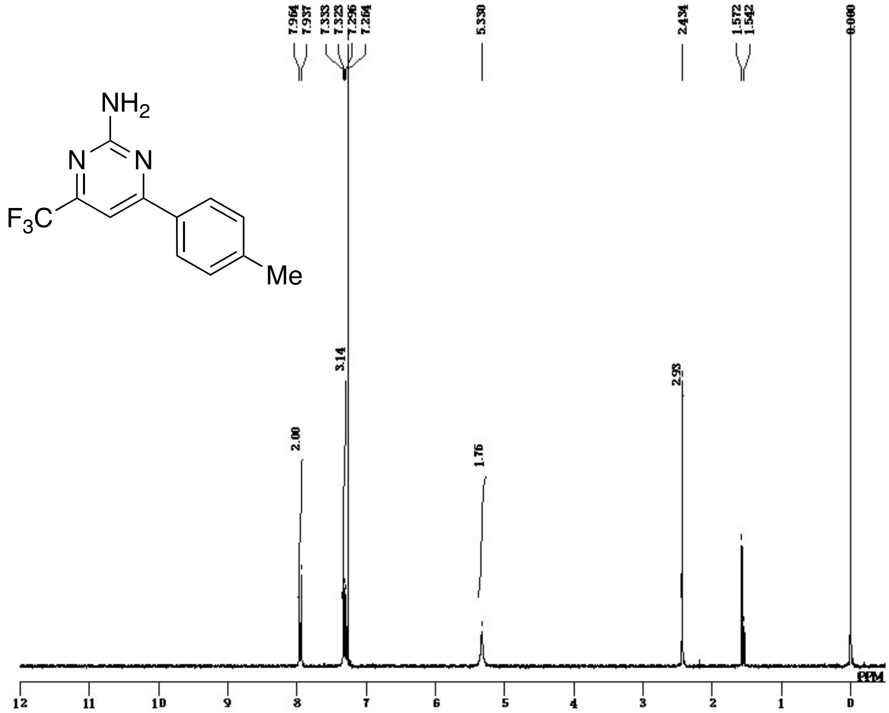

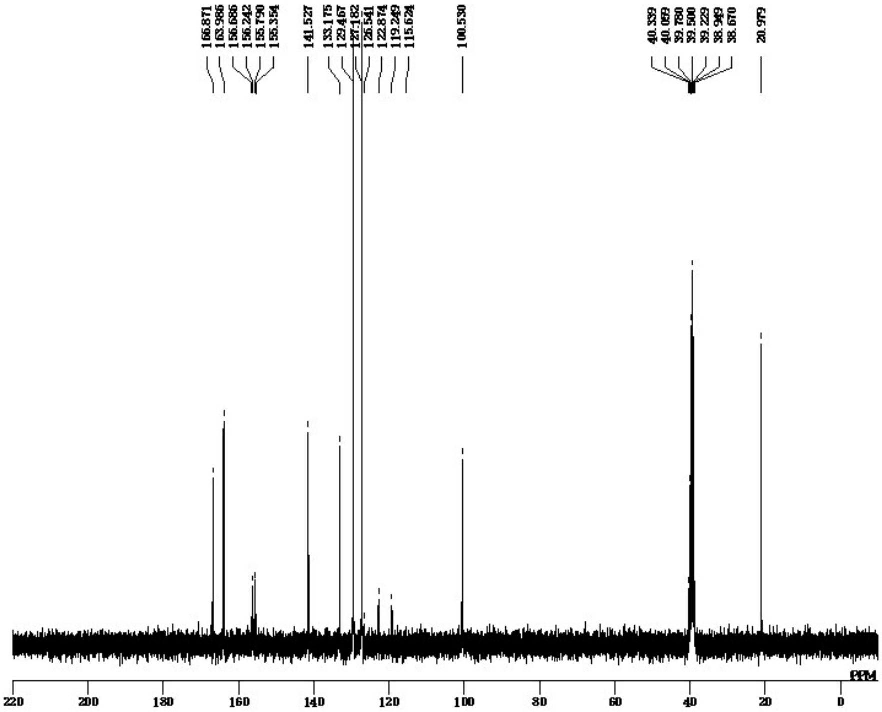

2-Amino-4-(4-bromophenyl)-6-(trifluoromethyl)pyrimidine (6ad)

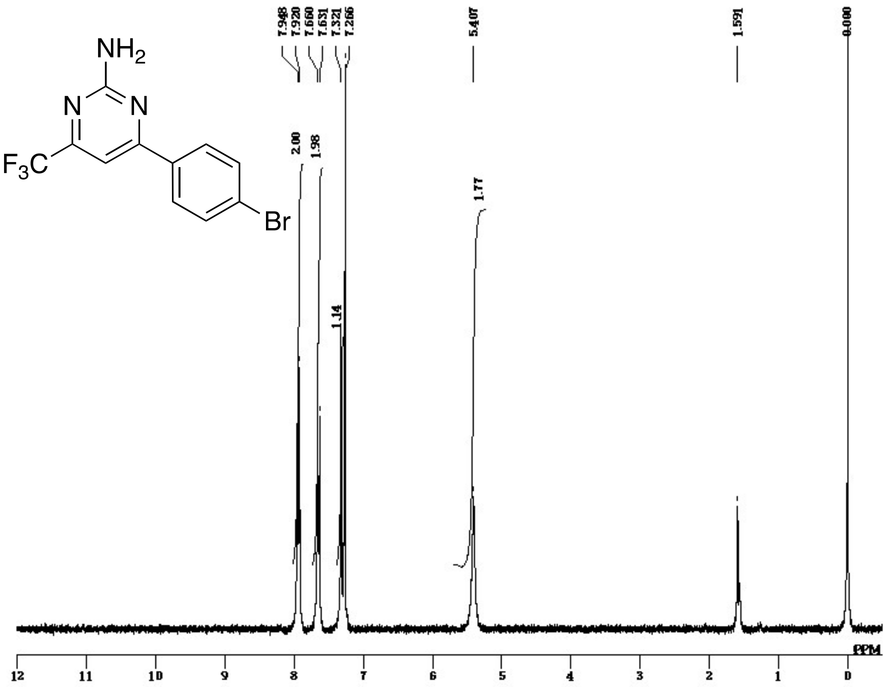

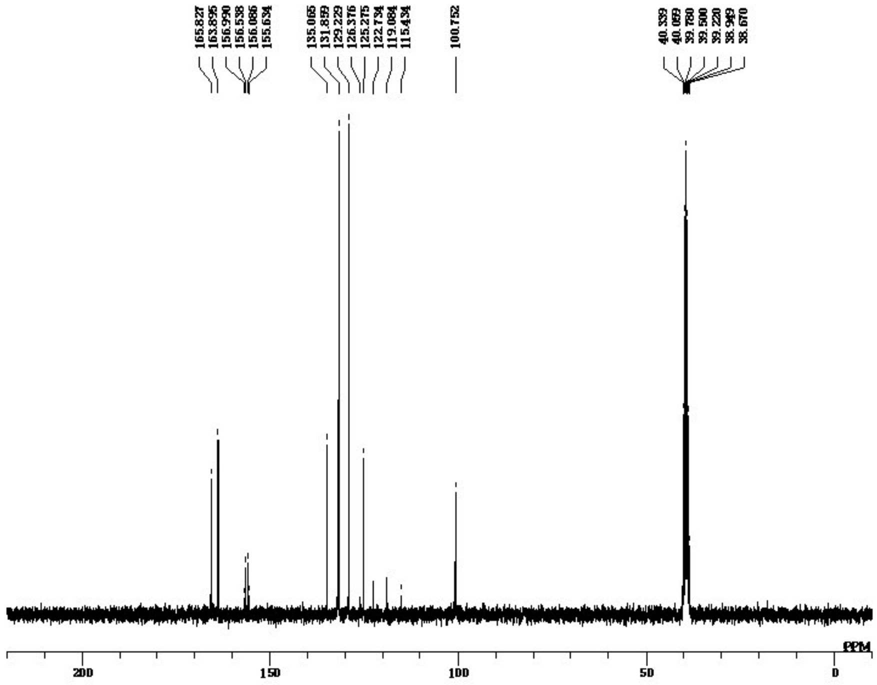

2-Methyl-4-phenyl-6-(trifluoromethyl)pyrimidine (6ba)

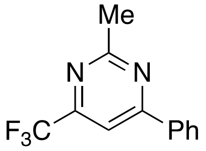

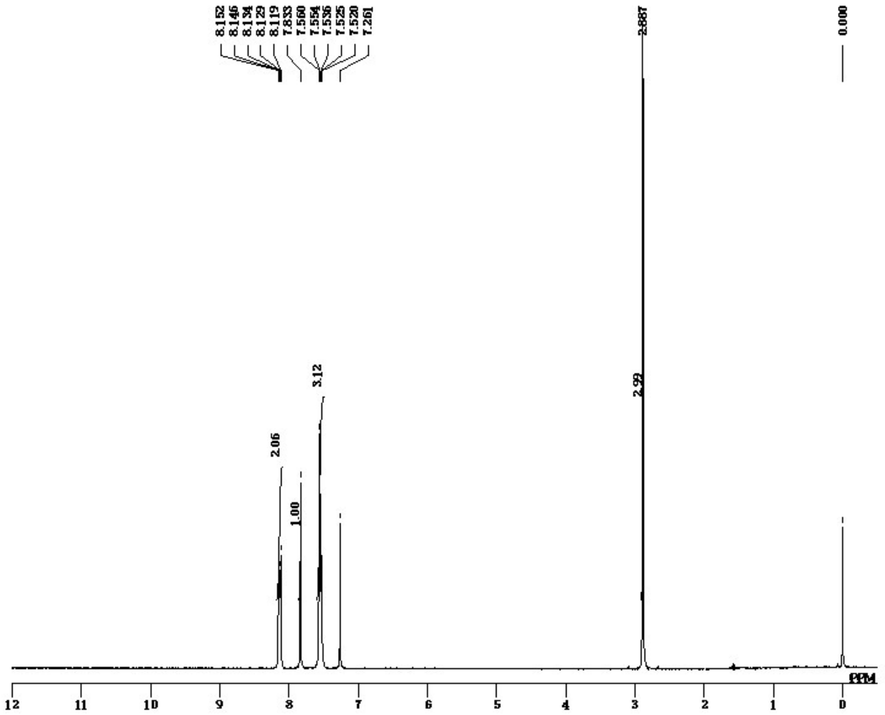

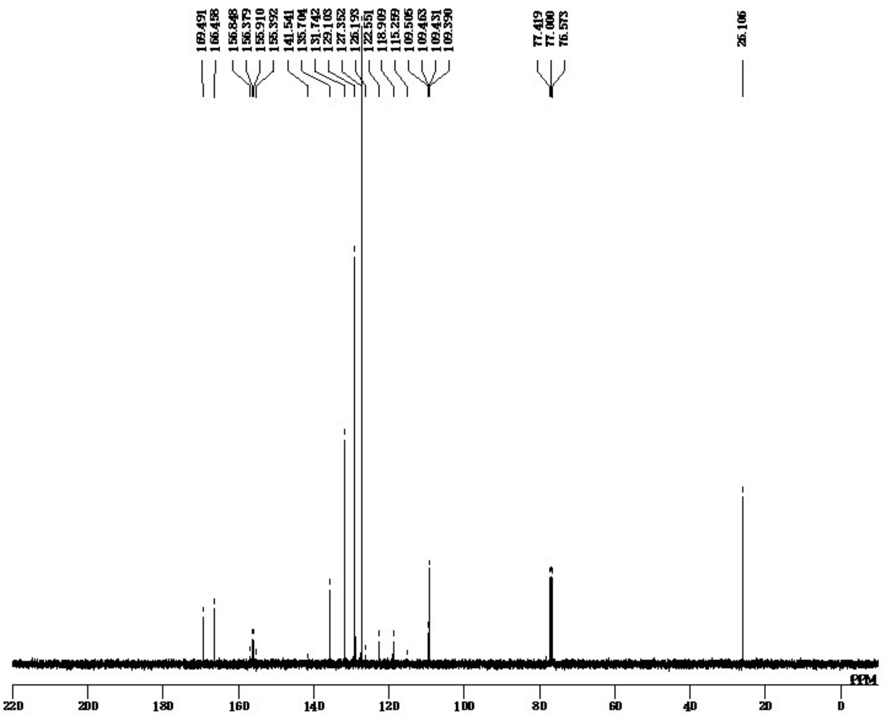

4-(4-Methoxyphenyl)-2-methyl-6-(trifluoromethyl)pyrimidine (6bb)

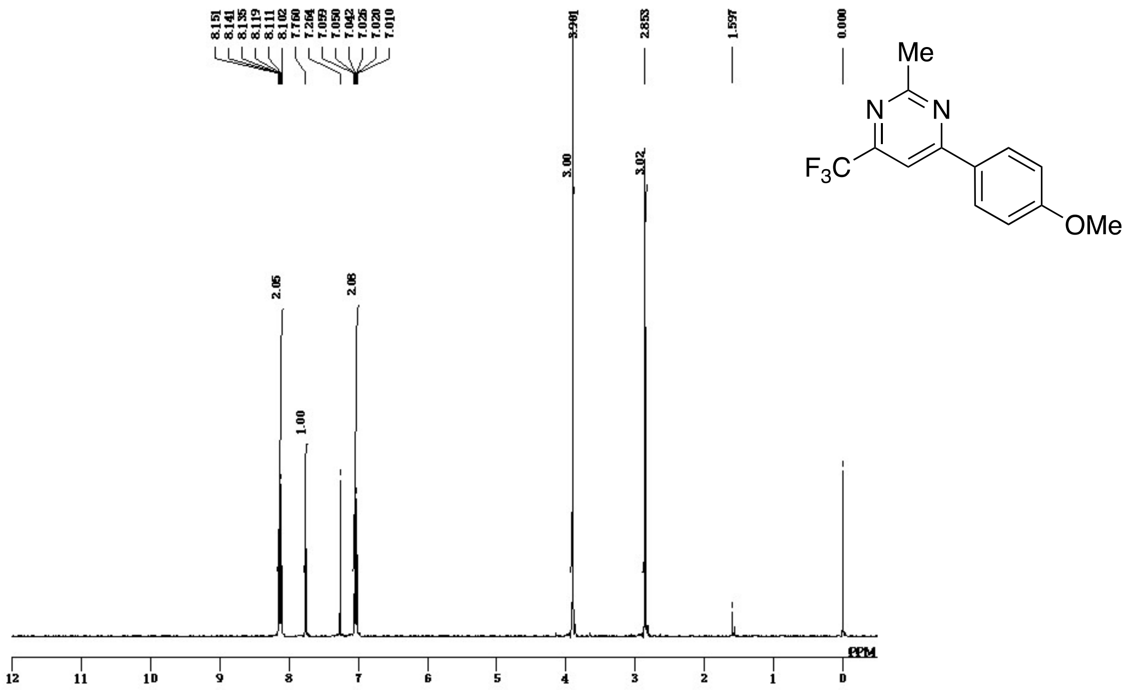

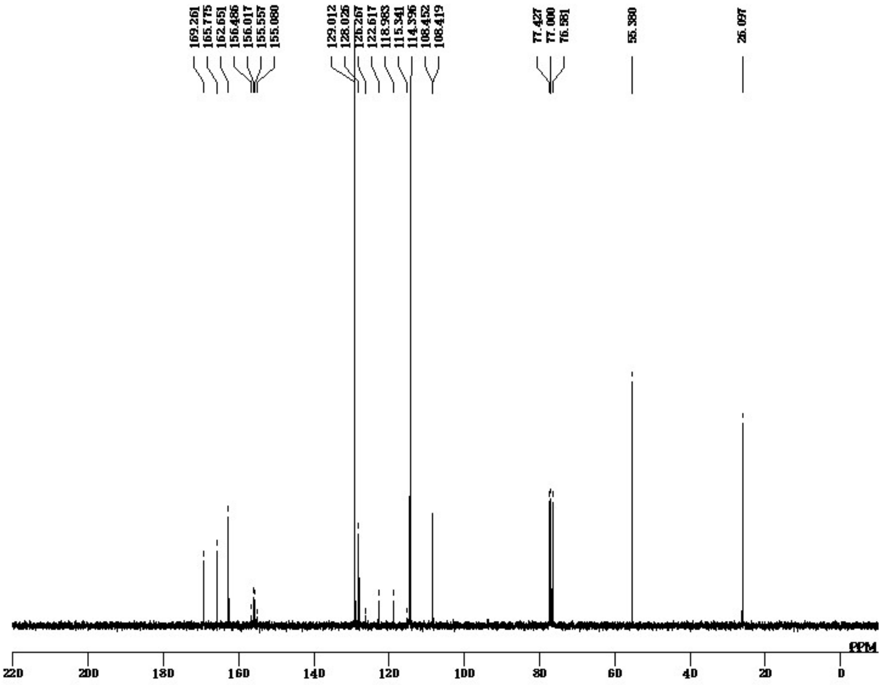

2-Methyl-4-(4-methylphenyl)-6-(trifluoromethyl)pyrimidine (6bc)

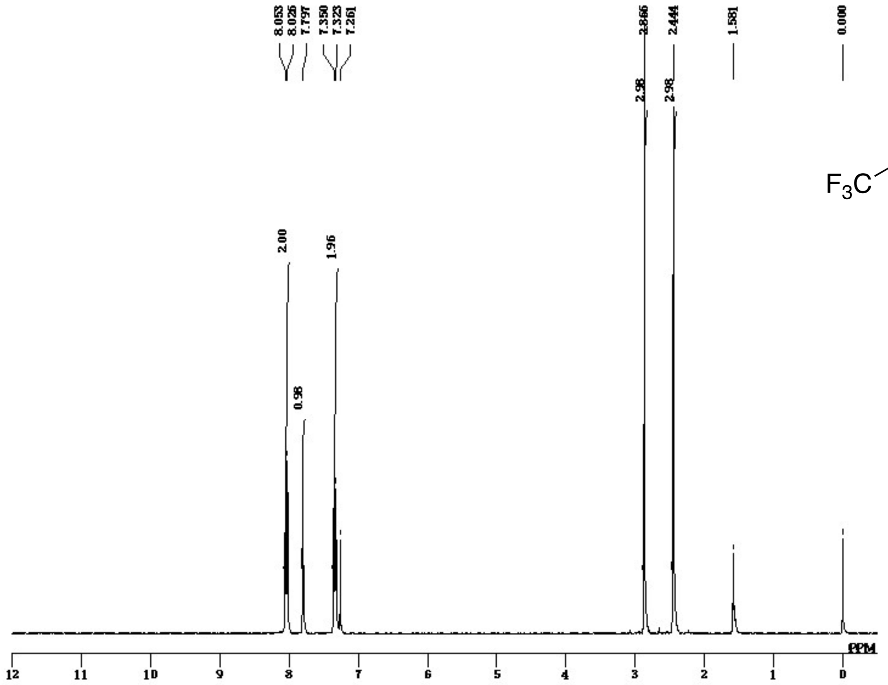

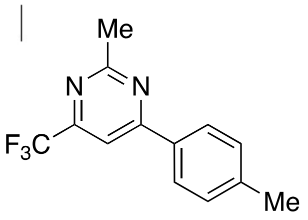

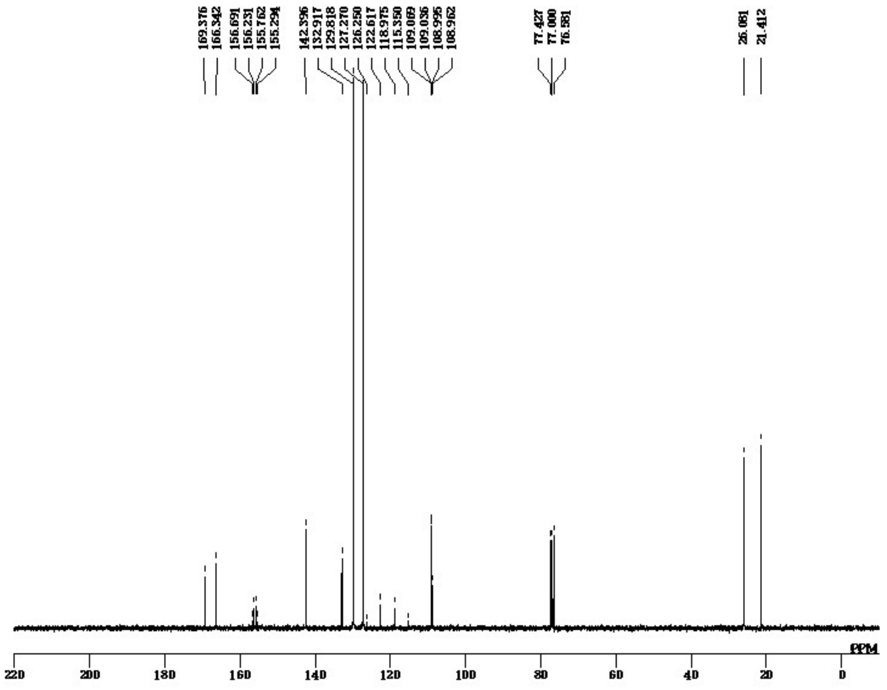

4-(4-Bromophenyl)-2-methyl-6-(trifluoromethyl)-pyrimidine (6bd)

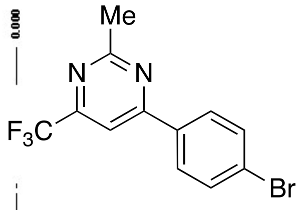

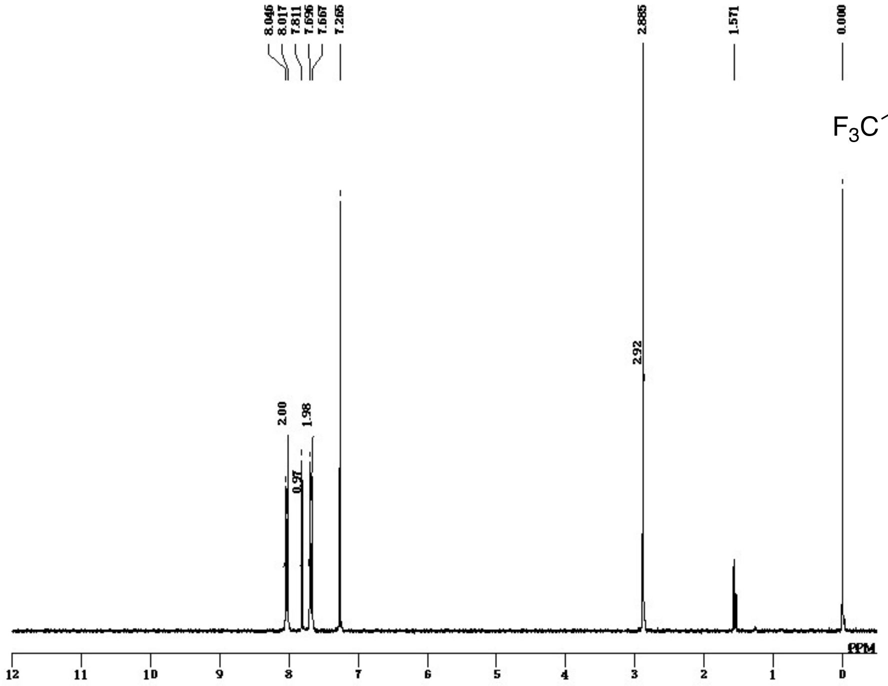

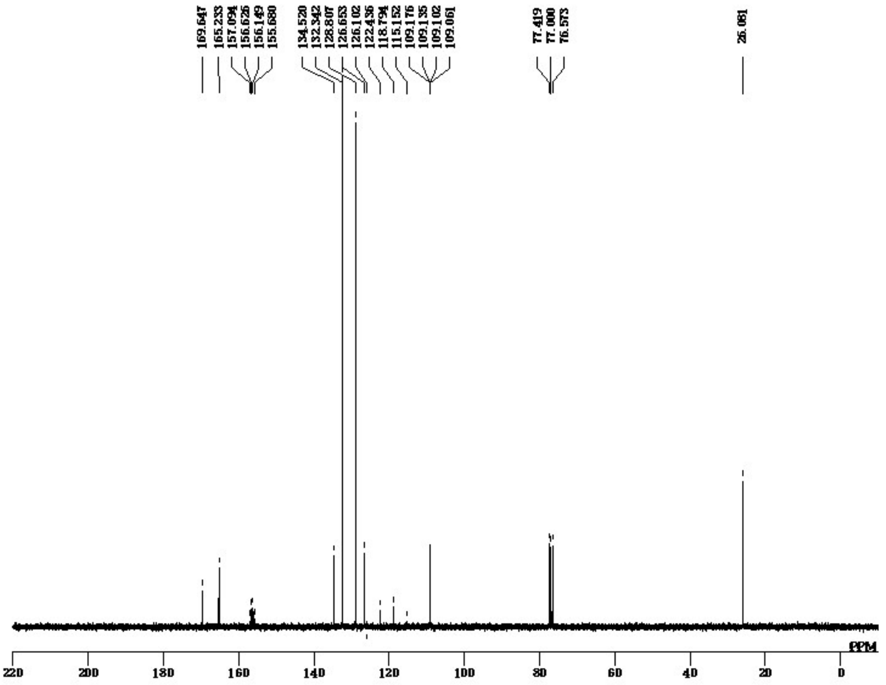

4-Phenyl-6-(trifluoromethyl)pyrimidine (6ca)

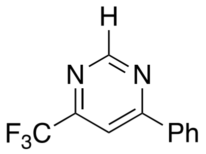

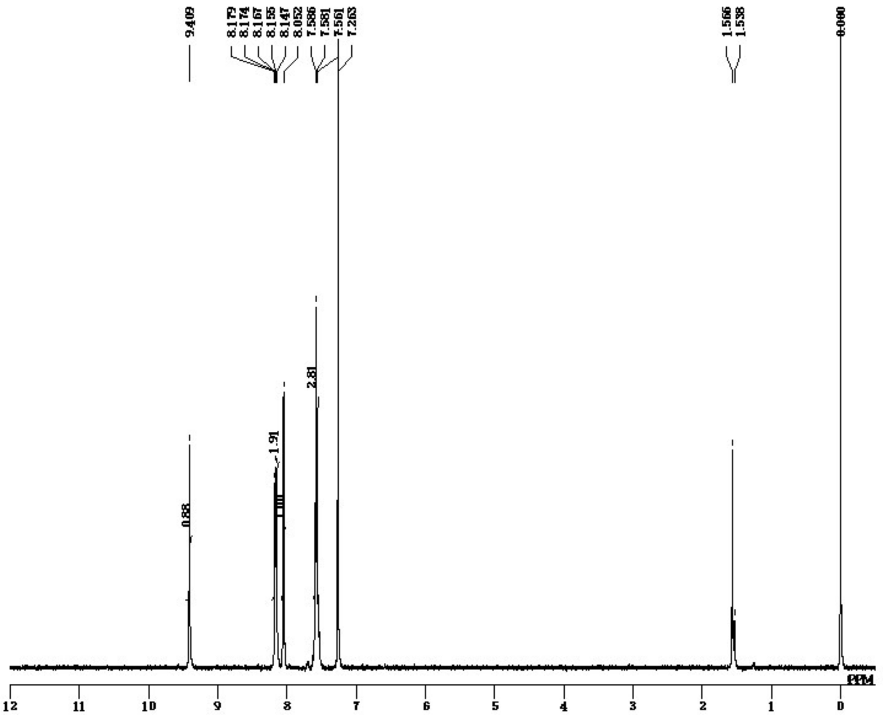

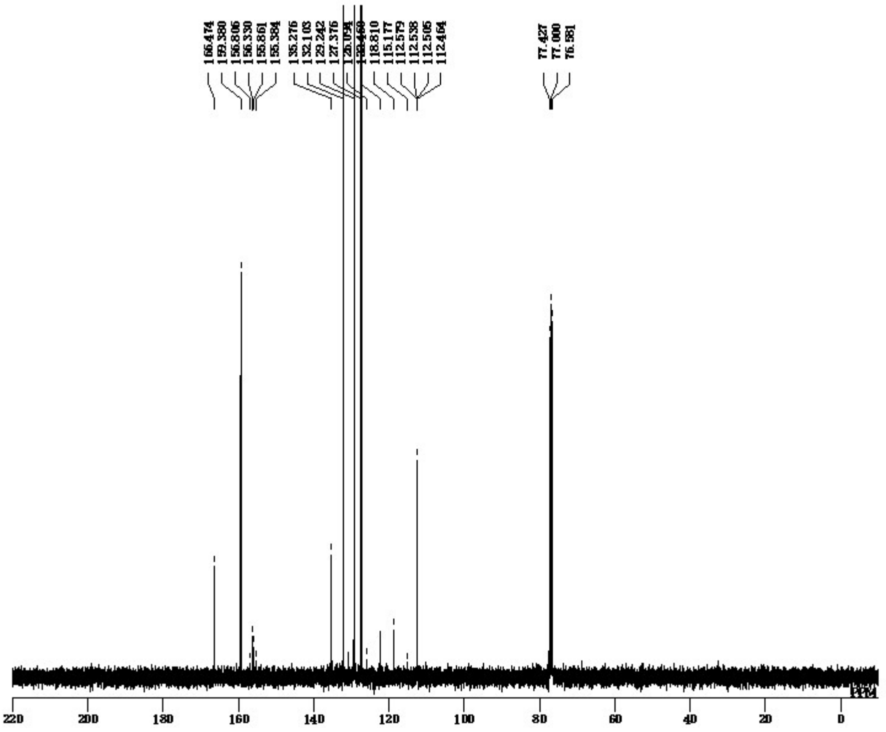

4-(4-Methoxyphenyl)-6-(trifluoromethyl)pyrimidine (6cb)

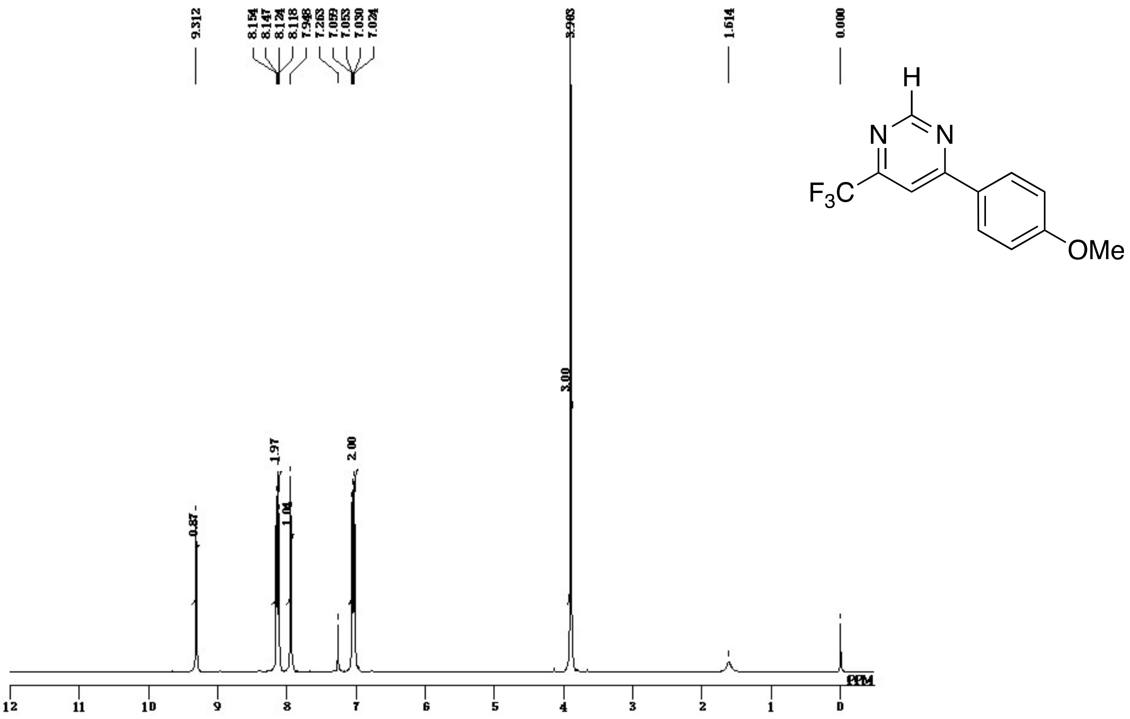

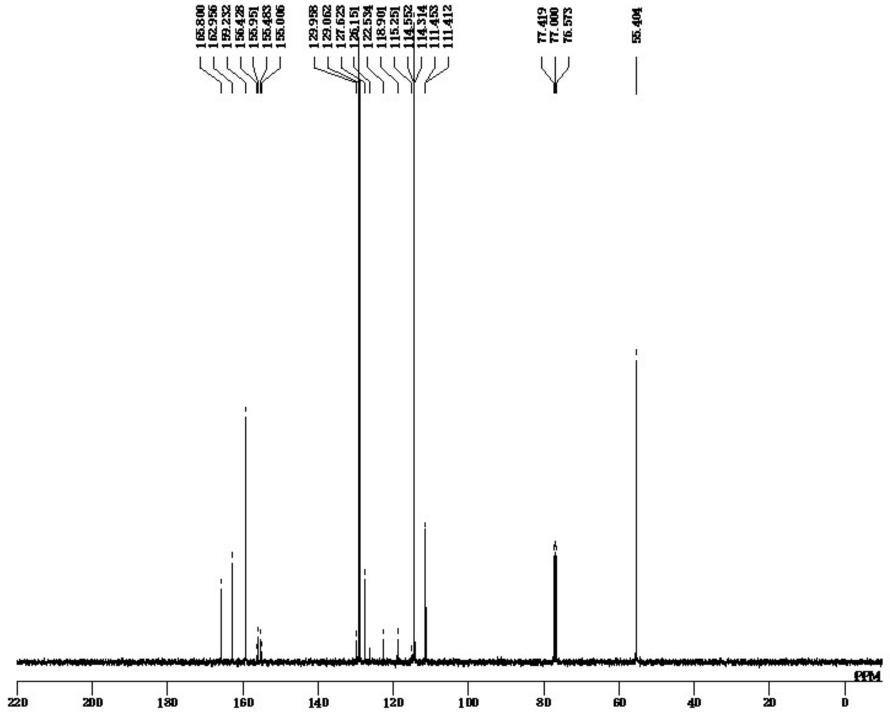

4-(4-Methylphenyl)-6-(trifluoromethyl)pyrimidine (6cc)

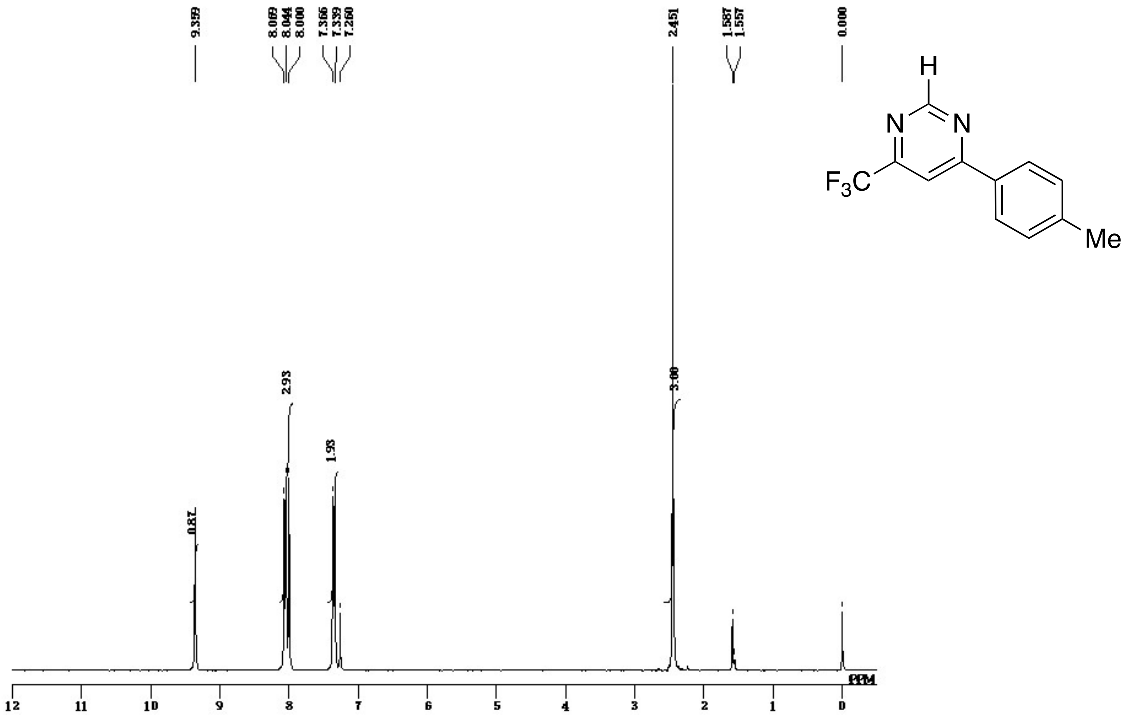

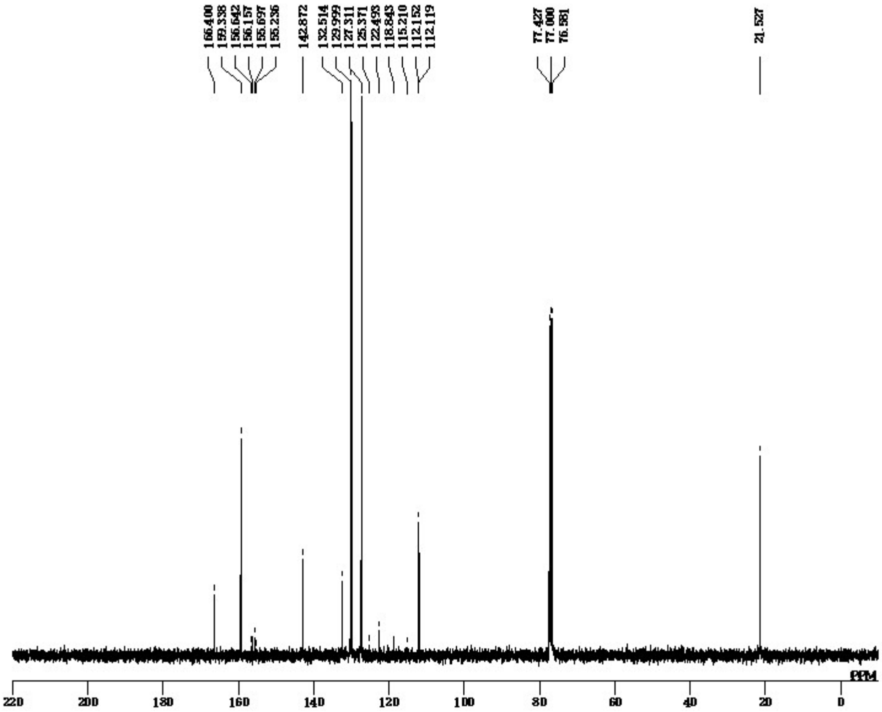

4-(4-Bromophenyl)-6-(trifluoromethyl)pyrimidine (6cd)

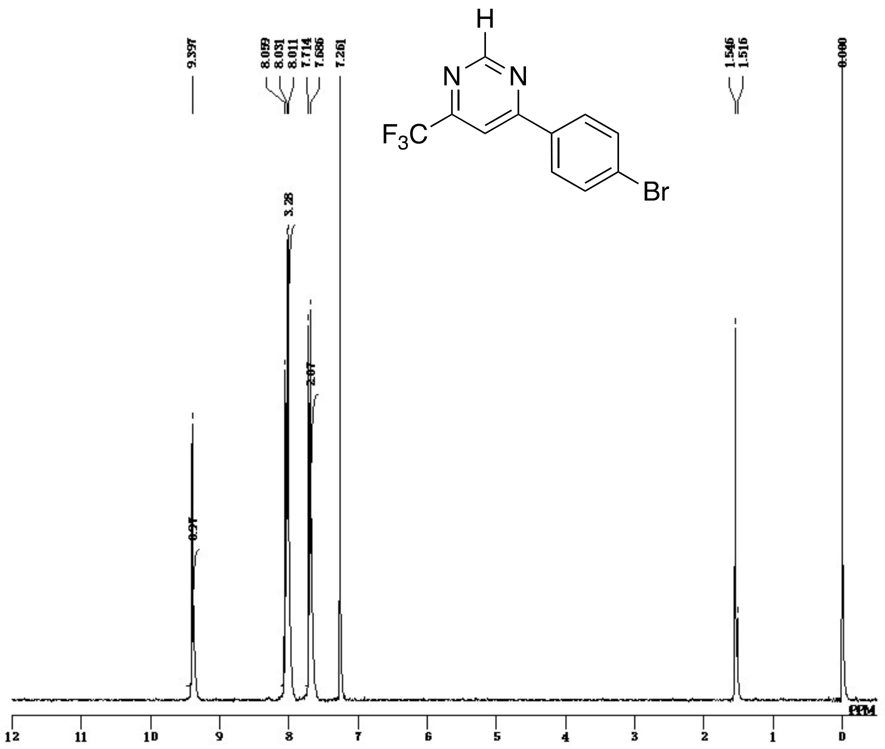

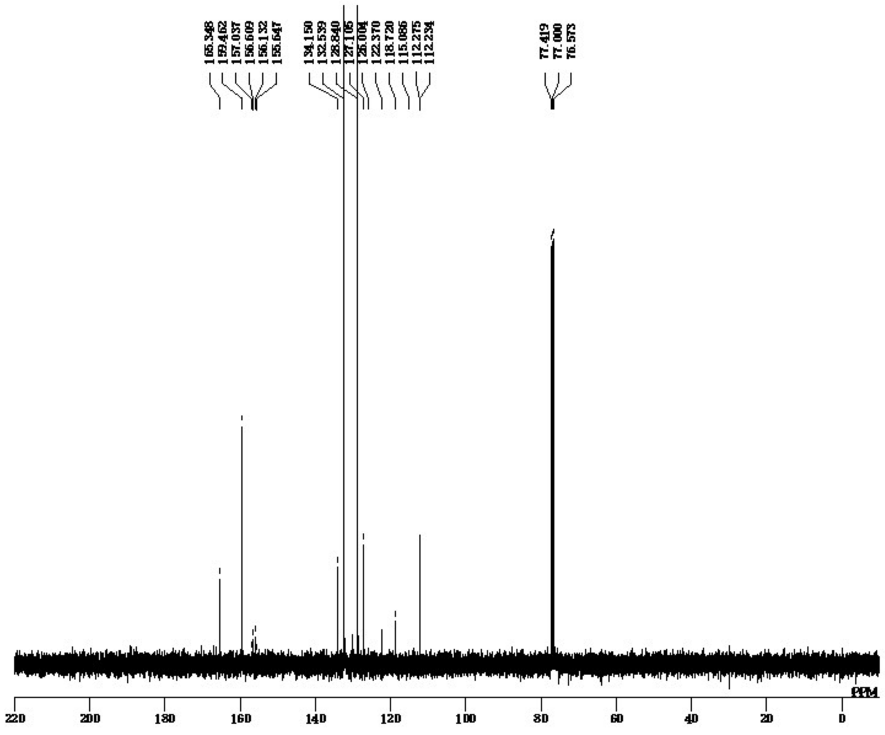

4. References

1. Shen, Y.-C.; Xin, Y.-K.; Cen, W.-B.; Huang, Y.-Z. *Synthesis* **1984**, 35–37.
2. Yamazaki, T.; Mizutani, K.; Kitazume, T. *J. Org. Chem.* **1995**, *60*, 6046–6056.
3. Miyagawa, M.; Naka, M.; Yamazaki, T.; Kawasaki-Takasuka, T. *Eur. J. Org. Chem.* **2009**, 4395–4399.
4. Yamazaki, S.; Yamamoto, Y.; Mikata, Y. *Tetrahedron* **2009**, *65*, 1988–1994.
5. Yamazaki, T.; Yamamoto, T.; Ichihara, R. *J. Org. Chem.* **2006**, *71*, 6251–6253.
6. Buettner, S.; Riahi, A.; Hussain, I.; Yawer, M. A.; Lubbe, M.; Villinger, A.; Reinke, H.; Fischer, C.; Langer, P. *Tetrahedron* **2009**, *65*, 2124–2135.
7. Fang, X.; Shen, G. Faming Zhuanli Shenqing (2012), CN 102675054 A 20120919.
8. Saijo, R.; Watanabe, G.; Kurihara, K.; Kawase, M. *Heterocycles* **2014**, *89*, 2334–2345.
9. Bonacorso, H. G.; Ferla, A.; Cechinel, C. A.; Zanatta, N.; Martins, M. A. P. *J. Heterocyclic Chem.* **2008**, *45*, 483–487.
10. Liu, C.-H.; Cui, Z.-N.; Yan, X.-J.; Qi, Z.-Q.; Ji, M.-S.; Li, X.-H. *Molecules* **2016**, *21*, 828/1–828/16.
11. Romanov, A. R.; Rulev, A. Yu.; Ushakov, I. A.; Muzalevskiy, V. M.; Nenajdenko, V. G. *Eur. J. Org. Chem.* **2017**, 4121–4129.
12. Romanov, A. R.; Rulev, A. Yu.; Popov, A. V.; Kondrashov, E. V.; Zinchenko, S. V. *Synthesis* **2020**, *52*, 1512–1522.
